# A Systems Model for Immune Cell Interactions Unravels the Mechanism of Inflammation in Human Skin

**DOI:** 10.1371/journal.pcbi.1001024

**Published:** 2010-12-02

**Authors:** Najl V. Valeyev, Christian Hundhausen, Yoshinori Umezawa, Nikolay V. Kotov, Gareth Williams, Alex Clop, Crysanthi Ainali, Christos Ouzounis, Sophia Tsoka, Frank O. Nestle

**Affiliations:** 1 St. John's Institute of Dermatology, King's College London, London, United Kingdom; 2 Biophysics and Bionics Lab, Department of Physics, Kazan State University, Kazan, Russia; 3 Wolfson Centre for Age-Related Diseases, King's College London, London, United Kingdom; 4 Department of Medical and Molecular Genetics, King's College London, London, United Kingdom; 5 Centre for Bioinformatics, Department of Computer Science, School of Physical Sciences & Engineering, King's College London, London, United Kingdom; Rutgers University, United States of America

## Abstract

Inflammation is characterized by altered cytokine levels produced by cell populations in a highly interdependent manner. To elucidate the mechanism of an inflammatory reaction, we have developed a mathematical model for immune cell interactions via the specific, dose-dependent cytokine production rates of cell populations. The model describes the criteria required for normal and pathological immune system responses and suggests that alterations in the cytokine production rates can lead to various stable levels which manifest themselves in different disease phenotypes. The model predicts that pairs of interacting immune cell populations can maintain homeostatic and elevated extracellular cytokine concentration levels, enabling them to operate as an immune system switch. The concept described here is developed in the context of psoriasis, an immune-mediated disease, but it can also offer mechanistic insights into other inflammatory pathologies as it explains how interactions between immune cell populations can lead to disease phenotypes.

## Author Summary

A functional immune system requires complex interactions among diverse cell types, mediated by a variety of cytokines. These interactions include phenomena such as positive and negative feedback loops that can be experimentally characterized by dose-dependent cytokine production measurements. However, any experimental approach is not only limited with regard to the number of cell-cell interactions that can be studied at a given time, but also does not have the capacity to assess or predict the overall immune response which is the result of complex interdependent immune cell interactions. Therefore, experimental data need to be viewed from a theoretical perspective allowing the quantitative modeling of immune cell interactions. Here, we propose a strategy for a quantitative description of multiple interactions between immune cell populations based on their cytokine production profiles. The model predicts that the modified feedback loop interactions can result in the appearance of alternative steady-states causing the switch-like immune system effect that is experimentally observed in pathologic phenotypes. Overall, the quantitative description of immune cell interactions via cytokine signaling reported here offers new insights into understanding and predicting normal and pathological immune system responses.

## Introduction

Inflammation is an organism's protective response to injury, pathogens or irritants and represents a complex multicomponent process that mobilizes immune cells to remove pathogens and restore tissue homeostasis. Healthy inflammatory reaction only lasts for a relatively short period of time, in contrast to pathological conditions where inflammation can persist over period of months or years. Chronic inflammation can be harmful and is attributed to the loss of balanced interactions between immune cells. Such interactions occur either via relatively small soluble proteins known as cytokines and chemokines, or through direct cellular interactions between ligands and their receptors expressed on the cellular surface [1]. Pathologies related to the immune system lead to a number of human diseases including psoriasis [2], arthritis [3], cancer [4], atherosclerosis [5], diabetes [6], inflammatory bowel disease [7], and asthma [8]. Even though each inflammation-mediated disease carries a set of unique features, a common trait between many inflammation-associated diseases is the chronic elevations of cytokine concentrations in the affected area.

**Figure 1:**
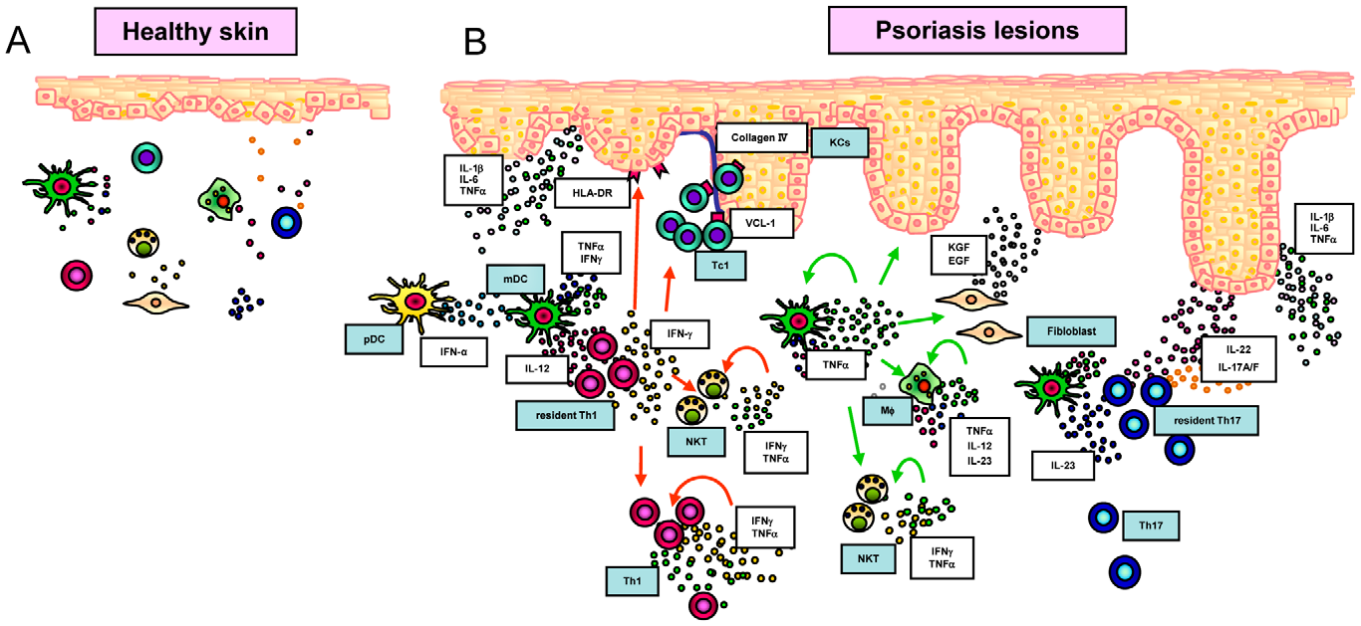
The schematic diagram for major cell populations involved in skin inflammation. A. Normal human skin contains a number of immune cells, including dendritic cells and macrophages that operate as sentinels. They are receptive to invading pathogens or other forms of physical, chemical or genetic damage. Upon activation, certain sub-populations of dendritic cells and macrophages attract and initiate numerous effector systems of the innate and adaptive immune systems. Locally activated immune system is characterised by inflamed tissue due to the increased cytokine concentrations. False activation of the immune system can lead to a number of pathologies, for example, psoriasis. B. Psoriasis is initiated by a number of factors such physical trauma, infection and drugs. The initial phase of developing psoriatic lesions is characterized by production of a large amount of IFN-γ by plasmacytoid dendritic cells (pDC). IFN-γ activates dermal myeloid dendritic cells (mDC) and initiates their migration to the local lymph node. In the lymph node mDCs induce proliferation and priming of antigen-specific T cells. mDC remaining in the dermis produce iNOS, IL-12, IL-23, and TNF-α proinflammatory cytokines. These cytokines initiate a chain of immune system reactions. The interactions between dendritic cells, lymphocytes and keratinocytes, create an area of persistent inflammation that can remain for a significant period of time. Human skin under inflammatory conditions contains increased numbers of immune cell populations and elevated levels of cytokines. The elevated concentrations of cytokines can remain for significant periods of time. While the same cells and elevated cytokine concentrations are observed in healthy skin, the major characteristic of pathology is the multifold increase of cell numbers and persistent maintenance of high cytokine concentrations. In response to inflammatory conditions keratinocytes undergo hyperproliferation and aberrant differentiation.

Skin is a preferred system for studying inflammatory conditions, as tissue can be both easily observed and sampled. Due to its easy accessibility it can be viewed as the “window” to the human immune system. Skin is composed of mainly two layers containing different cell types: keratinocytes are the major cell type forming the outer epidermis, whereas fibroblasts are the major component of the underlying dermis. In addition, various immune cells such as dendritic cell, T cells, neutrophils or natural killer cells reside in the skin and increase in number under inflammatory conditions [9]–[11]. Perturbations in the local immune system are found to be essential factors mediating skin disease [2]. Psoriasis is a chronic inflammatory skin disorder in which keratinocytes proliferate at an unusually rapid rate. The disease affects about 0.6–4.8% of the population [12] and is characterized by red, scaly patches that reveal fine silvery scales. Psoriasis usually develops on the knees, elbows and scalp, but can appear anywhere on the body [13]–[14]. Psoriasis serves as a good model for studies of inflammatory mechanisms and it is an attractive disease for proof-of-principle studies of new anti-inflammatory therapeutic strategies [15]. A schematic view of the role of the immune system in normal and inflamed skin is provided in Figure 1.

**Figure 2:**
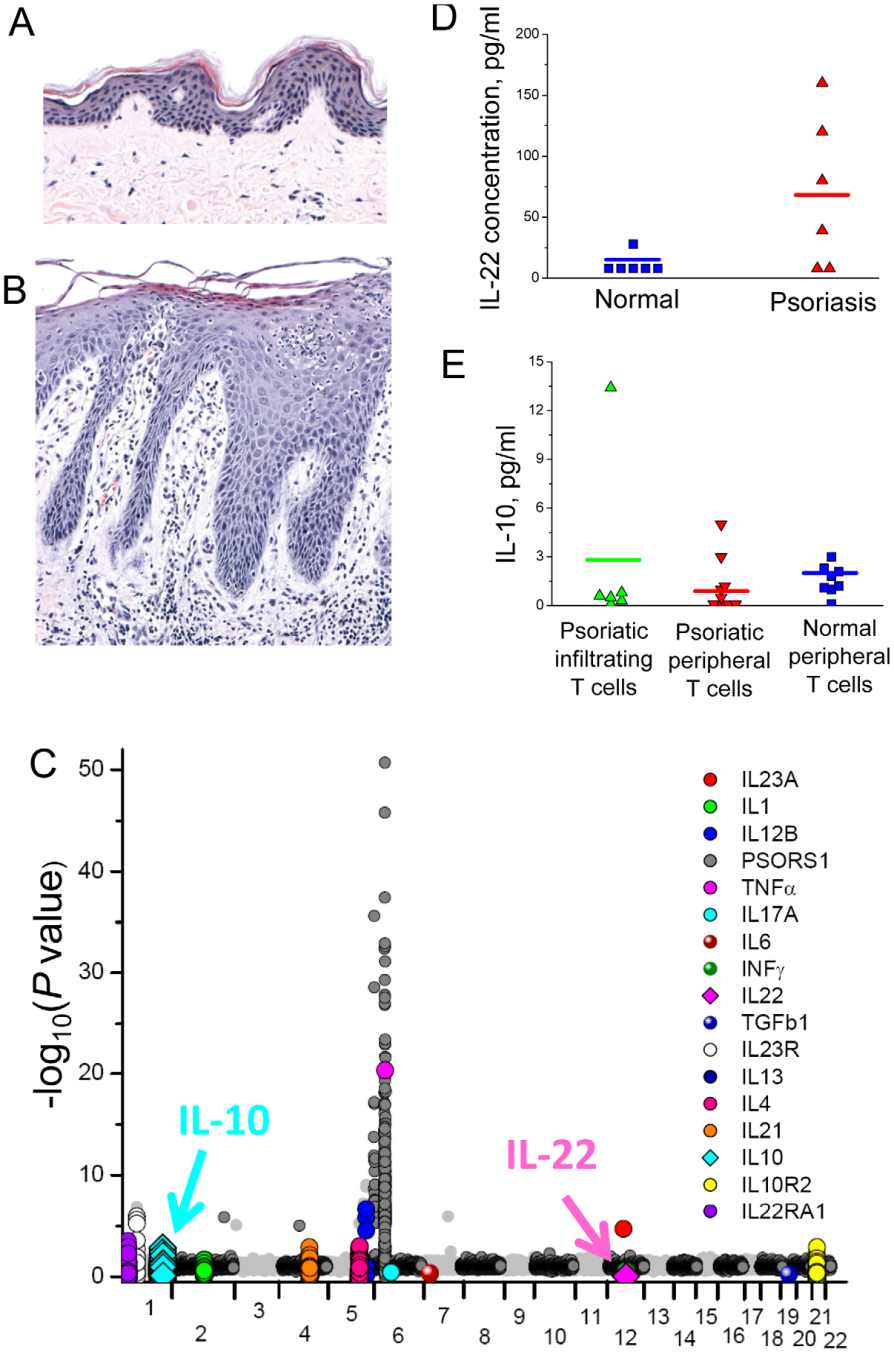
Comparison of normal and inflamed skin samples. Histology of psoriatic plaque (B) is compared with normal skin (A). Psoriatic plaque (B) is characterized by a hyperproliferative epidermal layer that contains a fourfold larger number of epidermal cells. C. Association significance for psoriasis is shown for the key inflammatory cytokines in the whole genome-wide context. It can be observed that the major cytokines IL22, INFγ, IL1, IL17A and IL6 cytokines do not meet the significance threshold. Genome-wide association of each SNP is plotted as the −log_10_ (*P*) dependence on the genomic location (in Mbp) using the coordinates of the NCBI Build 36.1 (March 2006). The association of the SNPs located within the 2 Mbp window centered at the selected inflammatory cytokines is shown in color for individual cytokines. D. The comparison of IL-22 concentration in healthy and psoriatic skin samples [37]. Although the genetic variant of IL-22 cytokine does not meet the association significance threshold (C), it is clearly present at higher concentrations in psoriatic skin samples. E. Opposite to the IL-22 example, the 2 Mbp region located at the IL-10 cytokine gene contains one of the 5% most significant SNP associated with psoriasis (C). However, the production of IL-10 does not significantly differ in healthy and inflamed skin [37]. The comparison of the association significance SNPs in the IL-22 and IL-10 cytokines and the actual cytokine concentrations in the skin shows that the GWAS and cytokine production/expression comparison between controls and cases may lead to conflicting conclusions.

A major histological feature of lesional psoriatic skin is the thickened epidermis which is due to hyperproliferation and abnormal differentiation of keratinocytes (Figure 2A and 2B). The increase in number of keratinocytes is about four-fold compared to normal skin [16]. The transition from normal to diseased skin has been shown to be dependent on immune cell infiltration into the dermis and epidermis (Figure 1) [2], [15]. Keratinocytes and immune cells interact via the release of soluble mediators such as cytokines and chemokines, as well as through cell-cell interactions mediated by surface-expressed ligands and receptors.

A widely held view is that psoriasis occurs as a result of unbalanced interactions between cells of the immune system, their mediators and keratinocytes [15]. Genetic studies have allowed the identification of a substantial number of loci harboring polymorphisms influencing the susceptibility to or protection from psoriasis. These studies are diverse and range from typing of serological variants of HLA-Cw6, to whole-genome linkage or association (GWAS) studies [17]. Although genetics studies validate the notion that key cytokine pathways are involved in the genetic susceptibility to psoriasis, they do not offer an explanation on how and why genetic variations in the cytokine mediated pathways lead to chronic inflammation. It is also unclear why in psoriasis some areas in skin are chronically inflamed, while others show no symptoms despite carrying the same disease-associated alleles. It is important to note that answering similar questions may be crucial in other inflammation-associated human conditions.

It has been suggested that chronic inflammation occurs as a result of a modified regulation in key immune cell populations via cytokine-mediated interactions. For example, T cells are reported to be regulated by dendritic cells via feedback control mechanisms [18], whereas Th17 cytokine mediated CCL20 expression in keratinocytes is implicated in psoriasis pathogenesis [19]. IL-21 has been shown to induce IL-17 through a self-amplifying loop [20]. IFN-γ and IL-17 secreted by activated CD4+ cells have been reported to up-regulate IL-6, IL-8, and CXCL10 production by benign prostatic hyperplasia cells [21], suggesting a positive feedback loop that amplifies inflammation in prostatic conditions. In another study, an example of the negative feedback loop in the NF-κβ-dependent cytokine pathway is reported to elevate the expression of proinflammatory cytokines [22]. Negative feedback control of the autoimmune response has been reported to occur through antigen-induced differentiation of IL-10-secreting Th1 cells [23]. Altogether, the above studies suggest that it is essential to investigate how immune cell populations switch from a healthy to a pathologic inflammatory response as a result of modified feedback interactions. Modified feedback loop interactions between immune cells in inflammation require the development of new computational strategies to describe how alterations in feedback loops relate to pathology.

A number of computational studies have offered insights into immune system signaling in the context of human disease. A model for a cell-cell interaction network has demonstrated that the loss of responsiveness in feedback signaling pathways is necessary and sufficient to induce leukemic transformation [24]. Immune system responses were evaluated for the tumor-immune system interactions by a mathematical model for melanoma invasion into healthy tissue [25]. It is reported that small metastatic lesions distal to the primary tumor mass can be held to a minimal size via the immune interaction with the larger primary tumor. A computational model has been used to determine the steady-state basal plasma glucose and insulin concentrations determined by their interaction in a feedback loop [26] and became one of the most well-recognized approaches for evaluating diabetes. Mathematical models developed to describe the dynamics of T cell homeostasis and proliferation were applied to provide insights into the CD4+ memory T cell depletion dynamics in HIV [27]. Other applications of translational systems biology in inflammation have been recently summarized in a comprehensive review [28].

These and other studies [29]–[34] have demonstrated that mathematical modeling can offer new insights into various aspects of inflammation by linking various experimental observations into an integrative model. However, the basic principles that distinguish healthy from pathologic inflammatory responses have not been elucidated or clearly explained yet. While it has been suggested that cytokine receptor polymorphisms can modify cytokine production by a small amount, there is currently no clear understanding of how such - seemingly insignificant - alterations can lead to disease. Experimental and computational studies need to lead to a framework that links genetic mutations to the (small) modifications of feedback loop interactions between immune cells which, in turn, may lead to pathology.

In order to address some of the outstanding questions and increase our understanding of how immune cell interactions contribute to normal or inflamed skin phenotypes, we developed a quantitative model that captures cytokine-dependent production profiles of cytokines in immune cell populations. The model represents the immune cell interactions as coupled cytokine concentration levels in human tissue by quantifying the underlying feedback loops. The approach allows the application of general concepts in dynamic systems modeling, such as stable homeostatic solution, feedback loops, bistability or oscillations, and thereby, uncovers the causes of chronic inflammation. Moreover, the methodology has the power to differentiate inflammatory disease phenotypes according to mechanisms of immune system imbalance. In this study we consider possible scenarios of cell population interactions and we show how even small changes in cytokine production rates by a single cell population can significantly affect systems properties due to altered feedback interactions and cause immune system-mediated pathology. The model also allows for discrimination between a healthy inflammatory response and chronic inflammation. Due to shared cytokine pathways between psoriasis and other chronic inflammatory diseases, the principles introduced in this study might be applicable to a wider range of immune system disorders.

## Results

### Experimental characterization of inflammation in human skin

Given the importance of cytokine-mediated interactions between immune cells, cytokine genes, gene products and their receptors have been subjected to genetic and immunological analysis. Cytokines form a group of candidate susceptibility genes in psoriasis [35]. For example, polymorphisms of the INF-γ and IL-10 genes were shown to be associated with different levels of cytokine production in patients with psoriasis [35]. Psoriasis is associated with over-expression of T-helper cell type 1 (Th1) cytokines, IFN-γ and TNF-α in the involved skin and relative underexpression of T-helper cell type 2 (Th2) cytokines, interleukin IL-4 and IL-10 [36]. Currently, the analysis of cytokine-mediated inflammatory conditions is performed on the bases of genetic association or case-control studies (GWAS) in combination with cytokine or expression production measurements. Frequently used causative indicators of disease occurrence are (i) disease-associated single nucleotide polymorphisms (SNPs) in cytokines and (ii) differentially expressed cytokine levels.

To evaluate the genetic association approaches and altered cytokine levels observed in psoriasis, we examined the degree of genetic association in polymorphisms located in the vicinity of key psoriasis cytokines. We re-analyzed the genetic association data obtained from GWAS for psoriasis [17]. In the Manhattan plot (Figure 2C), associations are highlighted corresponding to SNPs located in the genomic vicinity of a number of genes for key inflammatory cytokines crucial in psoriasis The Figure shows that none of the polymorphisms near the major cytokines IL-22, INF-γ, IL-1, IL-17A and IL-6 reached the significance association levels (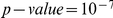) determined by the GWAS [17]. Since all these cytokines are shown to participate in the mechanism that mediates psoriasis [15], this result suggests that genotyping experiments do not represent an infallible method for identifying key pathology-associated cytokines.

To further assess the predictive capabilities of genome-wide screens for marker identification in inflammatory conditions, the differences in the IL-10 and IL-22 production levels between psoriatic and healthy skin samples were compared using experimental data from the literature [37]. We found that IL-10 is significantly associated with psoriasis GWAS [17] whereas IL-22 is not (Figure 2C). A comparison of the IL-22 concentration in the normal and psoriatic skin shows a significant elevation of IL-22 levels in disease [37] (Figure 2D), despite of the lack of significant IL-22 SNP in GWAS [17] (Figure 2C). The IL-10 cytokine shows the opposite effect to IL-22, as the SNP observed in the vicinity of the IL-10 gene shows a clear genomic association with psoriasis [17] (Figure 2C). At the same time, the IL-10 production by lymphocytes does not change significantly between cases and controls [37] (Figure 2E). Therefore, IL-10 and IL-22 cytokines are examples to demonstrate that either presence or absence of a SNP in a cytokine does not always translate to modified cytokine levels in pathological tissue. More specifically, the IL-10 cytokine example illustrates the case where a significantly associated SNP found in the cytokine does not result in altered cytokine levels, while the IL-22 example shows difference between cases and controls production levels in the absence of any significance in the genome wide scan.

The above examples suggest that although GWAS and cytokine production/expression comparison allow identification of potential cytokine candidates, they may lead to conflicting conclusions and do not establish a specific cytokine function. Moreover, one can argue that even in situations when both genetic significance and cytokine production/expression differences between cases and controls are present, the mechanisms of molecular interaction between immune cell populations in normal and pathologic interactions cannot be ascertained. It is also unclear how statistically significant differences for cytokines in genotype or expression data of disease and control cases contribute to unbalanced interactions between the immune cell populations. Therefore, the need exists for the development of additional methodologies complementary to genome-wide association studies and expression level comparison that would provide further insights into how the immune system operates.

### Interpretation of the cytokine production differences in the context of inflammatory disease

Genetic or expression level comparison studies are frequently complemented by cytokine concentration profiles, whereby the amounts of various cytokines produced by a specific cell population under normal and diseased conditions are measured by Luminex or Elisa assays. These techniques provide a closer insight into cellular interactions in disease, as individual SNPs or altered cytokine expression levels may not always translate into changes in cytokine production levels. In the previous section we showed that SNPs in cytokine genes may not always result in the modification of cytokine production profile.

In this section we demonstrate that up- or down-regulation of cytokine production levels in disease is due to the interactions between immune cells. Experimental measurements of cytokine production profiles in individual cell populations are usually performed in a physiological “cocktail” of other cytokines. Here we demonstrate that a random choice of the cytokine concentrations in such a physiological cocktail creates grounds for misconceiving the role of a particular cytokine in disease, as illustrated below.

**Figure 3:**
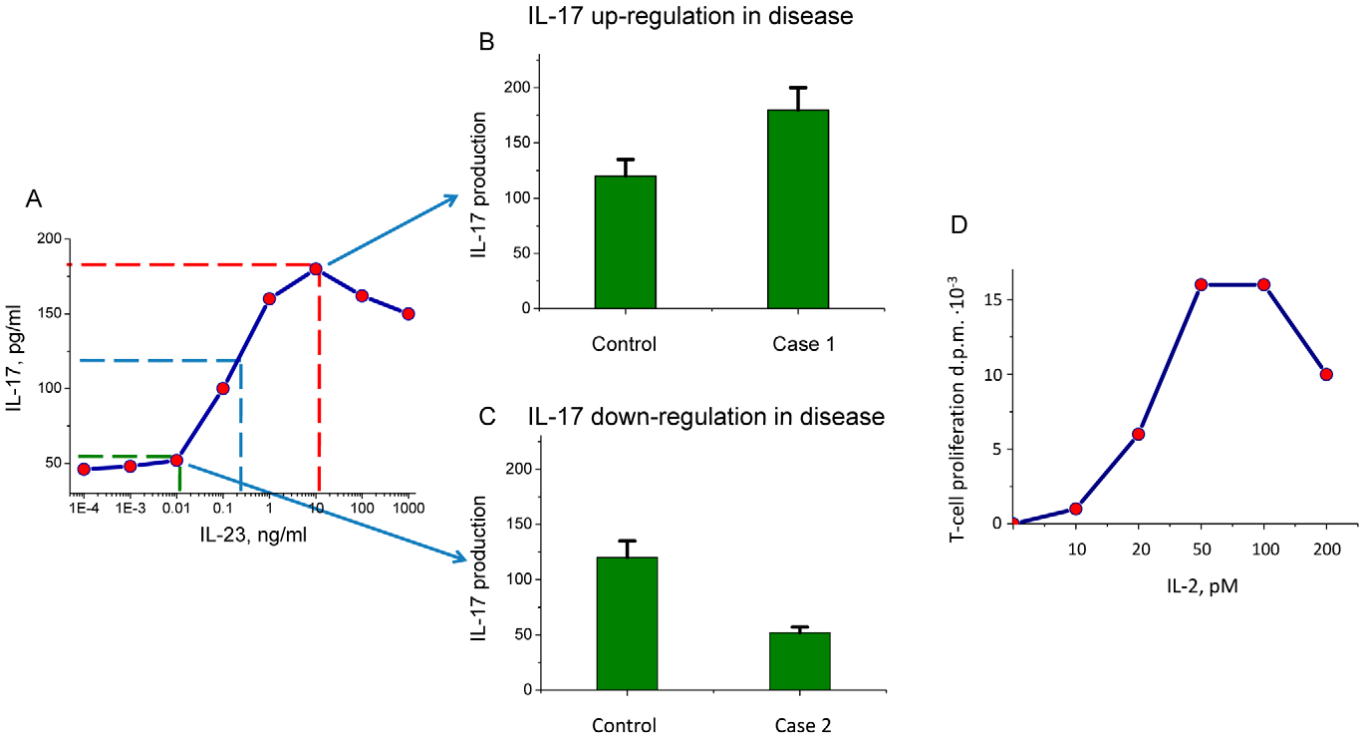
The dose-dependent production of IL-17 cytokine as a function of extracellular IL-23 concentration. Cytokine production rates by immune cell populations are measured at physiological, but often random background cytokine concentrations. This example illustrates that arbitrary choice of the background cytokine cocktail conditions (in this case IL-23) may lead to different IL-17 production results. A. IL-17 production by bone-derived marrow fibroblasts depends on IL-23 concentration, both important pro-inflammatory cytokines (concentration profiles adopted from [38]). Three random choices of background IL-23 concentration within the physiological range are indicated by dotted lines together with the relevant IL-17 concentrations. The possibility of conflicting IL-17 roles in disease is demonstrated by a comparison of higher (B) and lower (C) IL-17 production rates in the same cell population which is not attributed to either statistically significant SNPs in IL-17 or IL-23 genes or statistically significant alteration of cytokine expression levels. Instead, randomly chosen concentrations of physiologically important cytokines result in such dramatically different conclusions for the roles of pro- or anti-inflammatory cytokines. D. The graph adopted from [42] shows the T-cell proliferation rate as a function of the IL-2 concentration. This example suggests that the dose-dependent cytokine production by an immune cell population in skin can be due to the intrinsic cytokine-dependent properties of immune cells, but it is also modulated by dependence on proliferative and apoptotic phenomena on external cytokine concentrations in a dose-dependent manner.

Measurement of a particular cytokine concentration largely depends on the levels of other cytokines also present in the medium. We consider the dose-response curve for IL-17 production in bone marrow derived macrophages as a function of IL-23 concentration shown on Figure 3A, as adopted from [38]. Both IL-17 and IL-23 are major inflammatory cytokines, as identified by linkage analysis and functional characterization in a number of inflammatory conditions [13], [39]–[41]. The data show that IL-17 production has a complex dependence on extracellular IL-23 concentration. For example, the blue dotted line in Figure 3A indicates that for IL-23 concentration of 0.25 ng/ml, IL-17 provides a 120 pg/ml readout, while 10 ng/ml of IL-23 produces ∼180 pg/ml of IL-17. Therefore, variability in the IL-23 concentration within the physiological range is likely to cause significantly a different IL-17 production profile. The dotted green and red lines in Figure 3A indicate how the background concentration of IL-23 in the experimental medium can lead to either “upregulation” (Figure 3B) or “downregulation” effects in IL-17 production in disease even in the absence of any changes in bone marrow derived macrophage cytokine production properties (Figure 3C). This example illustrates that cytokine production profiles in immune cell populations cannot define the disease unambiguously and may lead to misinterpretation of cytokine production differences in control and disease samples (Figure 3).

It is essential to note that the overall cytokine production dependence in tissue combines both the cytokine production by a specific cell population as well as other cytokine-dependent effects, such as proliferation and apoptosis. Regulation through proliferation and apoptosis changes the number of cells in skin and therefore also modulates the dose-response profiles. For example, Figure 3D (adopted from [42]), shows the proliferation-apoptosis cycle of a T cell population with increasing IL-2 concentrations. Larger T cell pools produce greater amounts of cytokines and chemokines, therefore the total amount of cytokine production is by the cell numbers in Il-2 dependent manner.

### Definition of the homeostatic cytokine concentration

**Figure 4:**
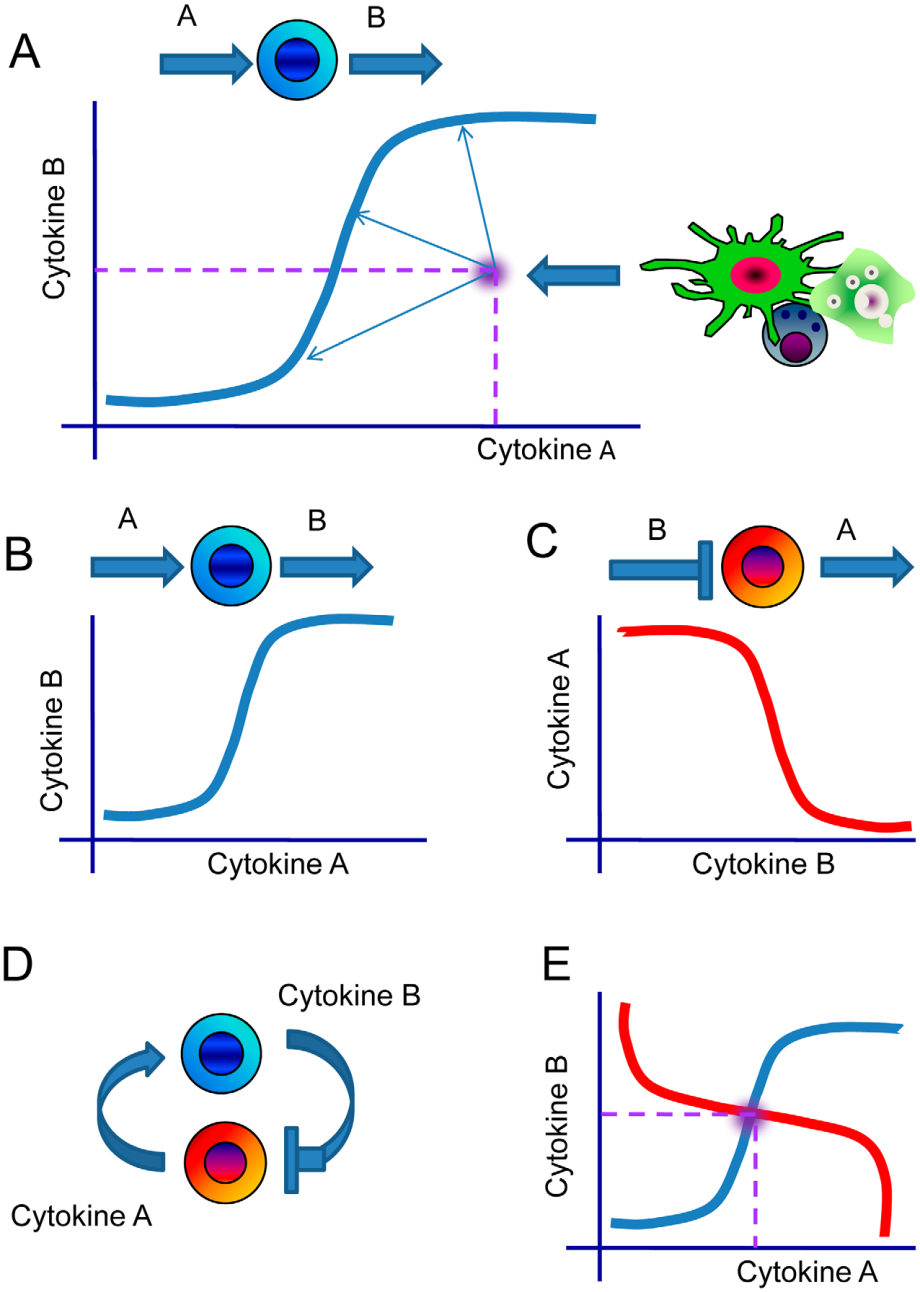
Dose-dependent cytokine production defines the homeostatic cytokine concentration. A. Cytokine B production by a cell population as a function of cytokine A concentration defines a continuous line of “homeostasis” for a given immune cell population, where for each concentration of cytokine A corresponds concentration of cytokine B produced by the specified cells. However, other immune cells in the tissue can also produce either or both cytokines A and B so that the combination of the A and B cytokine concentrations in tissue is no longer on the “homeostasis” line. Under normal conditions, the system has to return to a point of homeostasis on the “homeostatic” line. The point of homeostasis can be unambiguously defined by the superimposition of two interacting cell populations: one population produces cytokine B and has receptors to cytokine A (B) and another produces cytokine B in cytokine A-dependent manner (C). Simultaneous consideration of two interdependent cell populations (D) with the superimposition of the cytokine-cytokine dose-dependent curves (E) shows that the point of crossing is the only point where the homeostasis is achieved both “opposite” cell populations.

Cytokine production in a cell population is complemented by a number of mechanisms that counterbalance cytokine production in tissues. Extracellular concentrations of cytokines are affected by diffusion, cleavage by metalloproteases and cytokine binding followed by uptake. The dose-dependence of cytokine B on cytokine A concentration represents a dose-dependent curve of homeostatic balance. It is mediated by immune cell populations and balanced by the cytokine removal mechanisms described above. According to the dose-response curve, any given extracellular concentration of cytokine A in tissue translates to a specific extracellular concentration of cytokine B, under conditions of equilibrium. However, it is also possible that additional cytokine A or B production by other cell populations can also occur in tissue, resulting in cytokine A and B concentrations that do not fit the line of homeostatic equilibrium for the immune cell population considered (Figure 4A). After such perturbation, the immune system returns to homeostasis, defined as the dose-dependent line of cytokine B production in a cytokine A-dependent manner and modulated by the cytokine removal mechanisms. As shown in Figure 4A, there is an infinite number of homeostatic cytokine A and B concentrations that the system can adopt as it returns to equilibrium.

Owing to the infinite combination of cytokine A and B concentrations in homeostasis mentioned above (Figure 4A), at least two interdependent cell populations need to be considered to establish the conditions required for a specific homeostatic equilibrium. One immune cell population produces cytokine B in a cytokine A-dependent manner as previously described (Figure 4B) and the other cell population produces cytokine A in a cytokine B dose-dependent manner, where the cytokine B is chosen to have an inhibitory effect to the second cell population (Figure 4C). Both dose-dependent cytokine production curves represent the lines of homeostatic equilibrium for two “opposite” cell populations. The intersection of the two dose-dependent cytokine production profiles represents the point of synergistic balance, where both cell populations reach a homeostatic equilibrium. Such mutual dependence of cytokine concentration via the immune cell populations creates the classical problem of two interdependent variables that has been extensively studied in life sciences, but insufficiently recognized in immunology to-date. Such system-level effects can be associated with the presence of numerous interdependent cytokine pairs, whereby the interdependence can arise from either direct cell-to-cell interactions or larger number of interacting cell populations. Therefore, understanding of the immune cell interactions is enhanced by studying the experimental data through a quantitative description of cytokine production by cell populations in a cytokine-dependent manner.

Physiologically relevant consideration of two cell populations jointly (Figure 4D), suggests that the intersection of the dose-dependent curves occurs at a specific point, as shown in Figure 4E. This intersection defines the cytokine A and B concentrations unambiguously, as this is the only point where both cell populations reach homeostasis in equilibrium. Therefore, homeostatic cytokine concentrations can be defined as the extracellular cytokine concentrations where the immune system remains in equilibrium in the absence of normal or pathologic inflammatory response. From a systems perspective, the inflammatory response can be defined as the system response to the temporally perturbed shift from equilibrium with the ensuing return to homeostasis.

### Cytokine homeostasis in the absence of inflammatory pathology

**Figure 5:**
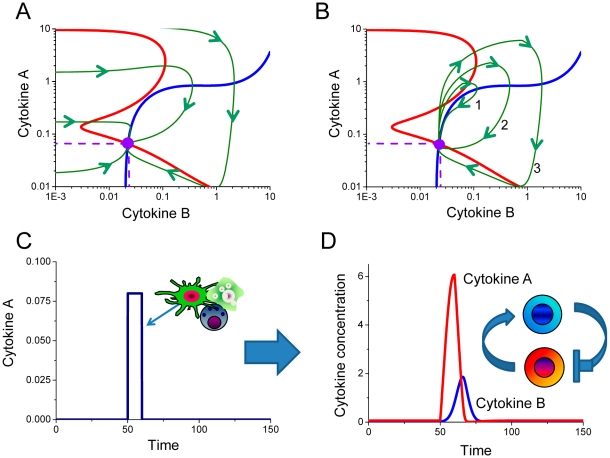
Systems model predictions for immune sub-system response containing two interdependent immune cell populations. A. Phase diagram shows simulated lines (red and blue) of homeostasis for two cell populations. The intersection of the cytokine dose-dependent curves defines the stable steady-state solution that represents the homeostatic concentrations for cytokines A and B as indicated by the violet dotted lines. The green lines describe the trajectories of A and B cytokine alterations from any non-homeostatic combinations of A and B concentrations. Arrowheads indicate the directions of the cytokine concentration alterations towards the homeostatic point of equilibrium from any nonequilibrium combination of cytokine concentrations, as predicted by the systems model. B. When an immune system in homeostasis is exposed to external or internal temporal cytokine application, it responds by generating a cytokine impulse. The response of interdependent cell population to small external perturbation can be introduced by other immune cells. The green lines show the trajectories of cytokine concentration divergence from homeostasis in response to small and transient external cytokine A impulses. Trajectories 1 and 3 occur in response to smallest and largest cytokine A applications, respectively. C. The largest external perturbation leading to trajectory 3 on (B). D. The immune sub-system cytokine A and B spikes, generated in response to the external cytokine A spike on (C). The comparison of the impulse applied and the response generated shows clearly that a relatively small application of cytokine A can generate an impulse nearly two orders of magnitude larger compare to the applied spike. Such model prediction suggests that (i) an immune system can amplify inflammatory signals and (ii) even a healthy system can experience a significant, but transitory, elevation of cytokine concentrations above homeostatic levels.

In order to model the performance of the immune system under normal homeostatic conditions, we analyzed dynamic system responses shown in Figure 5. The phase diagram (Figure 5A) depicts two overlapping cytokine dose-response curves for two cell populations (red, blue curves) intersect at one point (violet circle). The dotted lines represent predicted homeostatic concentrations for cytokines A and B. The red and blue dose-response curves are defined as *null clines* or *lines of equilibrium*. Vector fields are also shown to represent the cytokine concentration dynamics at the non-equilibrium levels (Figure 5A).

It is noted that the cytokine dose-dependent relationships shown on Figure 4 are schematic and intended for illustrative purposes only, while Figure 5 describes the predictions of the mathematical model. According to the model, the dependence of cytokine A on cytokine B concentration (blue curve) represents a classical dose-dependent activation of one cytokine by another, while the reverse dependence of the cytokine B production as a function of cytokine A concentration (red line) reveals a significant nonlinearity. Mechanistically, such dependence can occur when the model parameters are set such that the cytokine production is nonlinearly related to the cytokine concentration-dependent uptake (please refer to the Materials and Methods section for the detailed description of the model and the underlying parameter values). At the same time, the highly nonlinear relationship between cytokine A and B concentrations (red curve) corresponds to the experimentally observed IL-17 production as a function of IL-23 concentration (Figure 3A) in bone-derived marrow fibroblasts [38]. The dose-dependent curve of IL-17 production as a function of IL-23 (Figure 3A) is “rotated” by 90° and superimposed on the dose-dependent curve for IL-23 production as a function of IL-17 concentration (Figure 5). While we believe that the proposed framework of immune cell interactions analysis is generic and applicable to various pairs of immune cell populations or pairs of cytokines, we note that cytokines IL-17 and IL-23 are good candidates to showcase the systems model presented in this manuscript.

The quantitative representation of immune cell interactions offers a number of mechanistic insights into the immune system responses, specifically in the activation dynamics in response to external application of cytokine A, applied at the state of homeostatic equilibrium (Figure 5B). Three cytokine dynamic profiles annotated as 1, 2 and 3 show the interconnected cell population responses to the temporal application of external cytokine A in increasing amplitude. In all three cases, both cytokine concentrations increase temporally and converge back into the same point of homeostasis. External perturbations of the highest amplitude that induced response 3 on Figure 5B applied to the system of two interacting cell populations are shown on Figure 5C. The temporal cytokine A and B dynamics in response to the small and temporal external perturbation by other immune cell populations (Figure 5C) or infection is presented on Figure 5D. This graph is a temporal projection of trajectory 3 from Figure 5B and clearly illustrates that a small external perturbation applied for a small duration induces cytokine A and B impulses of significantly higher amplitude and somewhat longer duration. The cytokine concentrations released into the extracellular space dramatically diminish in concentration as cytokine diffuses in all possible directions. Our model predicts that a normal immune system is very sensitive and capable of amplifying very small cytokine impulses followed by a return to the original level of homeostasis.

### Additional steady-state levels of homeostasis causes inflammatory disease

Inflammation-mediated skin conditions are characterized by chronically high cytokine concentrations maintained over extended periods of time. We employed our mathematical model to explore potential factors that can turn normal immune system responses into pathology.

To explore the ability of the model to predict pathologic immune responses, we varied parameter values (Table 1) of the governing equations in the model without changing the structure of equations used, to ensure that we simulate the same cell populations that could originally produce a normal immune response. The alteration of model parameter values reflects the influence of internal and environmental factors to the immune cell populations.

**Table 1:** Parameter values employed in the systems model for the immune cell interactions.

Parameter	Value	Figure №	Dynamic properties of the immune system
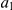	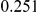	Figure 5	Stable Homeostasis
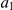	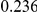	Figure 6	Trigger switch
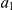	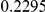	Figure 7, 8, 9	Oscillations
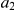	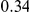	Figure 5	Stable Homeostasis
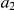	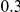	Figure 6	Trigger switch
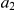	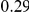	Figure 7, 8, 9	Oscillations
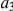	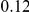	Figure 5	Stable Homeostasis
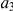	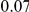	Figure 6	Trigger switch
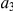	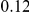	Figure 7, 8, 9	Oscillations
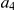	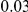	Figure 5	Stable Homeostasis
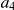	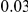	Figure 6	Trigger switch
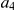	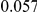	Figure 7, 8, 9	Oscillations
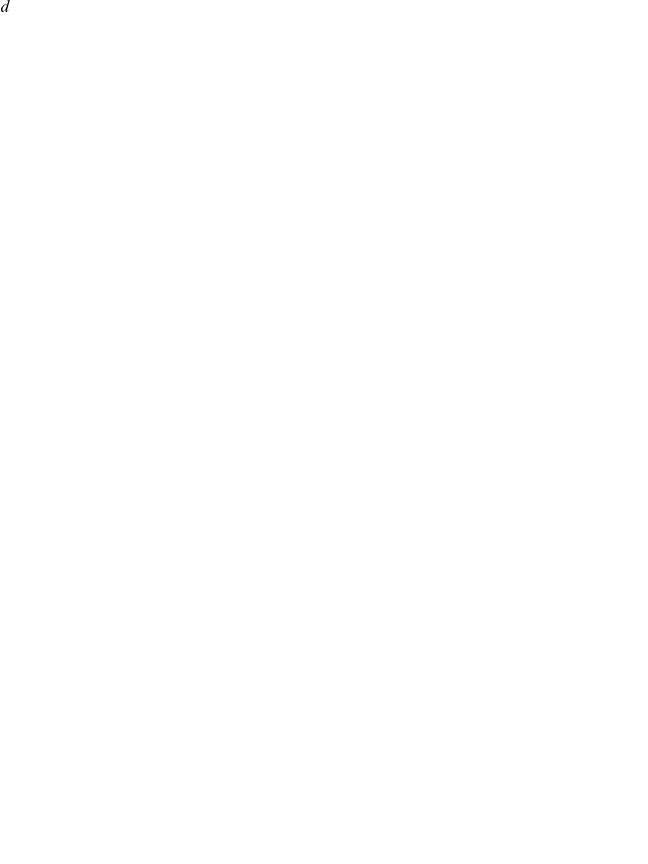	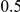		
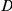	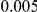		
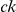	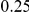	Figure 5	Stable Homeostasis
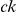	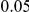	Figure 6	Trigger switch
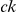	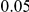	Figure 7, 8, 9	Oscillations
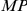	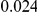		
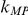	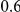		

**Figure 6:**
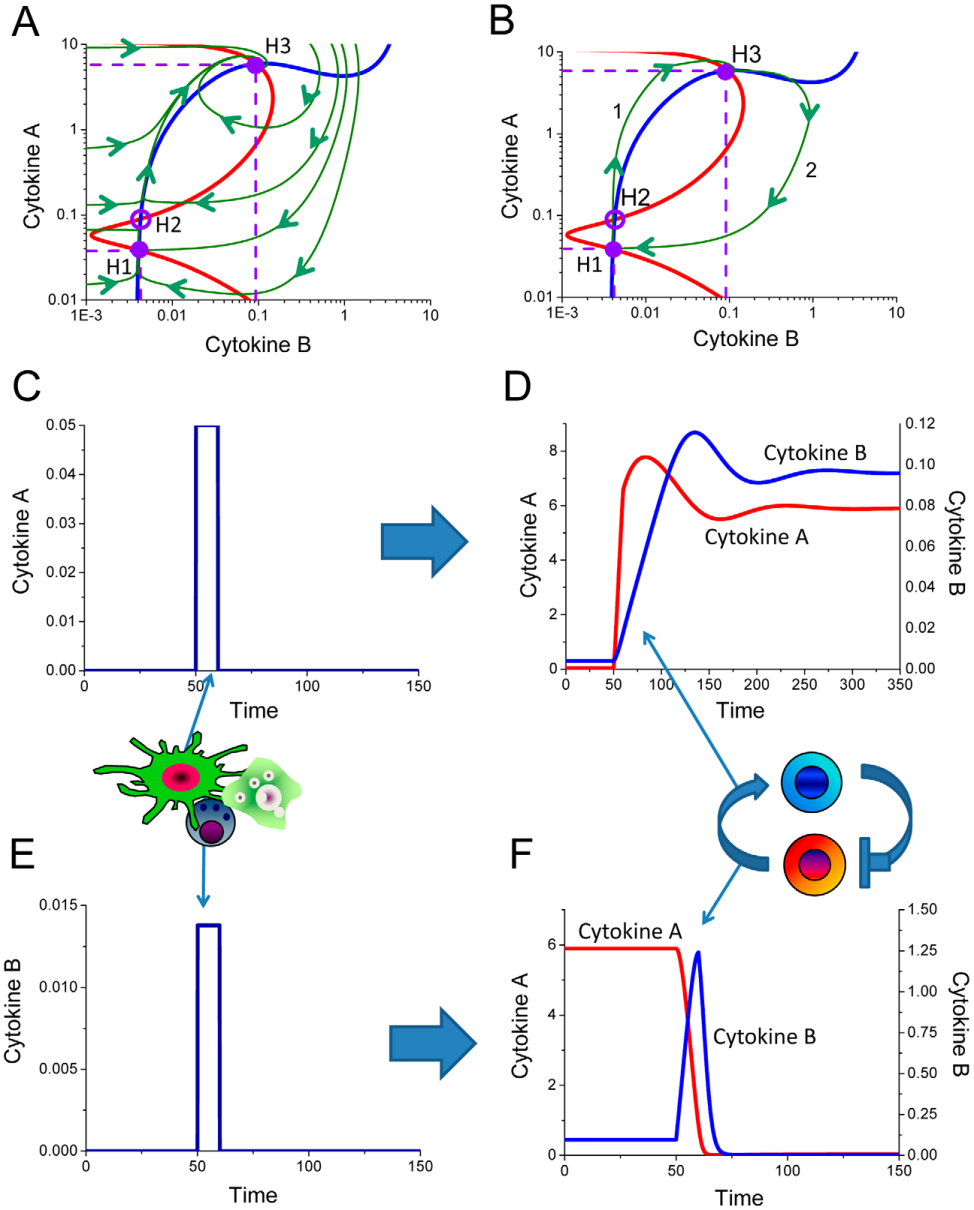
The systems model predicts the cytokine trigger dynamics. Internal or external factors can change the cytokine production profiles and thereby modify the immune cell interaction parameters via feedback loops. A. The nullcline diagram shows the possibility for two interacting immune cell populations to have multiple levels homeostasis as indicated by the intersections of the red and blue cytokine dose-response curves. The two filled circles represent stable solutions, whereas the hollow circle indicates the unstable solution. The green lines describe how the system converges into the stable homeostatic from any non-homeostatic combination of cytokine concentrations. B. The mathematical model predicts that an immune sub-system can switch between the states of stable low and high cytokine concentrations. The trajectory 1 shows the transition from the lower to higher homeostatic points in response to the external cytokine A impulse, whereas the transition from the higher to the lower homeostatic point occurs after the application of the external cytokine B as indicated by the trajectory 2. The time course of cytokine alterations during the transition from the low to high concentration states in response to the external cytokine A impulse (C) is shown on (D). The cytokine concentration dynamics during the switch from the higher to the lower homeostasis states in response to the external impulse of cytokine B (E) is shown on (F).

Figure 6A shows the nullclines that represent the dose-dependent cytokine production rates for two interacting cell populations. Since the underlying equations have not been modified and parameters have only been altered in a minor fashion, the shapes of the dose-dependent cytokine production profiles are similar to the ones predicted for a healthy immune system, shown on Figure 5A. However, slight modifications in the immune cell interaction parameters (that can be caused by genetic polymorphisms, environmental factors or a combination of the two) cause the nullclines to intersect at three different points (marked by violet circles and annotated as H1-H3): two intersections occur in the area of the low cytokine concentrations, and the third is observed at the region of significantly higher cytokine concentrations. Numerical simulations reveal that only two of the three solutions are stable (H1 and H3, shown as filled violet circles on Figure 6A), whereas the intermediate one is unstable (H2 indicated as a hollow violet circle). This suggests that two interacting immune cell populations can create more than one homeostatic level of cytokine concentration in extracellular space.

The described result implies that even minor alterations in cytokine production profiles (presumably initiated by a combination of genetic polymorphisms and environmental factors) can lead to pathological inflammation resulting from modification of feedback loop parameters. The model predicts that causative SNPs that contribute to the alteration of feedback loops via small modifications of cytokine profiles do not need to be identical across all disease phenotypes. The model relates SNPs to pathologic levels of cytokine concentration in tissue and predicts that both statistically significant and insignificant genetic polymorphisms from different immune cell populations can lead to the appearance of additional pathologic cytokine levels and describes how. This offers an explanation of why only some areas of skin can be inflamed in psoriasis while others exhibit symptomless phenotype, while all cells across the whole body carry the same genetic polymorphisms. The systems analysis indicates that genetic polymorphisms can operate in combination with external conditions and either lead to inflammation, or exhibit symptomless phenotype depending on the environmental stimulus. However, one can argue that feedback loop modifications between immune cell populations originates from genetic variants, which do not need to be the same in all disease states and can lead to the emergence of pathology with or without environmental factors.

The directed green lines on Figure 6A represent the vector field and show the dynamic cytokine trajectories that converge into one of the stable homeostatic solutions from any combination of cytokine concentrations. In this case, interacting cell populations can maintain two distinct homeostatic cytokine levels, one in the area with low and with high cytokine concentrations. The systems model predicts that under a certain combination of parameters, interacting cell populations are capable of operating as a switch that can shift between two distinct homeostatic levels of cytokine concentrations. The appearance of additional stable homeostatic solutions suggests that the immune system can remain in a state of elevated cytokine concentrations for a significant period of time. The existence of two stable solutions creates a different scenario than in the case where interactions between immune cells had only one single stable homeostatic solution. More specifically, in healthy immune system temporal elevation of cytokine concentrations are always followed by an imminent return to homeostatic concentrations. In this pathologic scenario, the alterations of cytokine concentrations can cause the immune system to return to either of two stable homeostatic levels; the state of low cytokine concentration or the pathologic one of high cytokine concentrations, where the immune system can remain for a significant duration. The possibility to switch between two stable cytokine concentration levels provides the trigger-like properties to the system of at least two immune cell populations.

Next, we analyzed the dynamics of cytokine alterations at the transition between the two stable homeostatic levels. Figure 6B shows the variations of cytokine as the system switches from the homeostatic point of low cytokine concentrations (H1) to the homeostasis point with high cytokine concentration (point H3, green trajectory 1) and back (green trajectory 2). Under the assumptions underlying the present model, the transition from H1 to H3 occurs upon external impulse of cytokine A (Figure 6C). The cytokine A and B alteration dynamics during the transition from H1 to H3 is shown on Figure 6D. The model predicts that the transition from H3 to H1 can be induced by application of external cytokine B (Figure 6E). The transition from chronically high cytokine concentrations to the low level is shown schematically on Figure 6F. The model predicts that cytokine B is capable of generating a significant spike before shifting to H1. The model predictions address the fundamental question of how lesional and perilesional skin phenotypes can simultaneously coexist in inflammatory condition affected patients. The trigger-like cytokine behavior emerging from the interactions between the cell populations can keep the skin either in the inflamed condition causing a lesion, or remain at the lower cytokine concentration steady-state level observed in perilesional skin samples.

### The loss of homeostatic stability induces a different disease phenotype

Psoriasis is characterized by a variety of clinical phenotypes. After establishing the mechanism of chronic inflammation in the form of additional stable homeostatic level as described previously, we employed the systems model for immune cell interactions to elucidate whether it can uncover the causes of variety of clinical phenotypes observed in clinical practice. Similarly to the previous case, we tested combinations of parameters within physiological limits without changing the structure of the governing equations.

**Figure 7:**
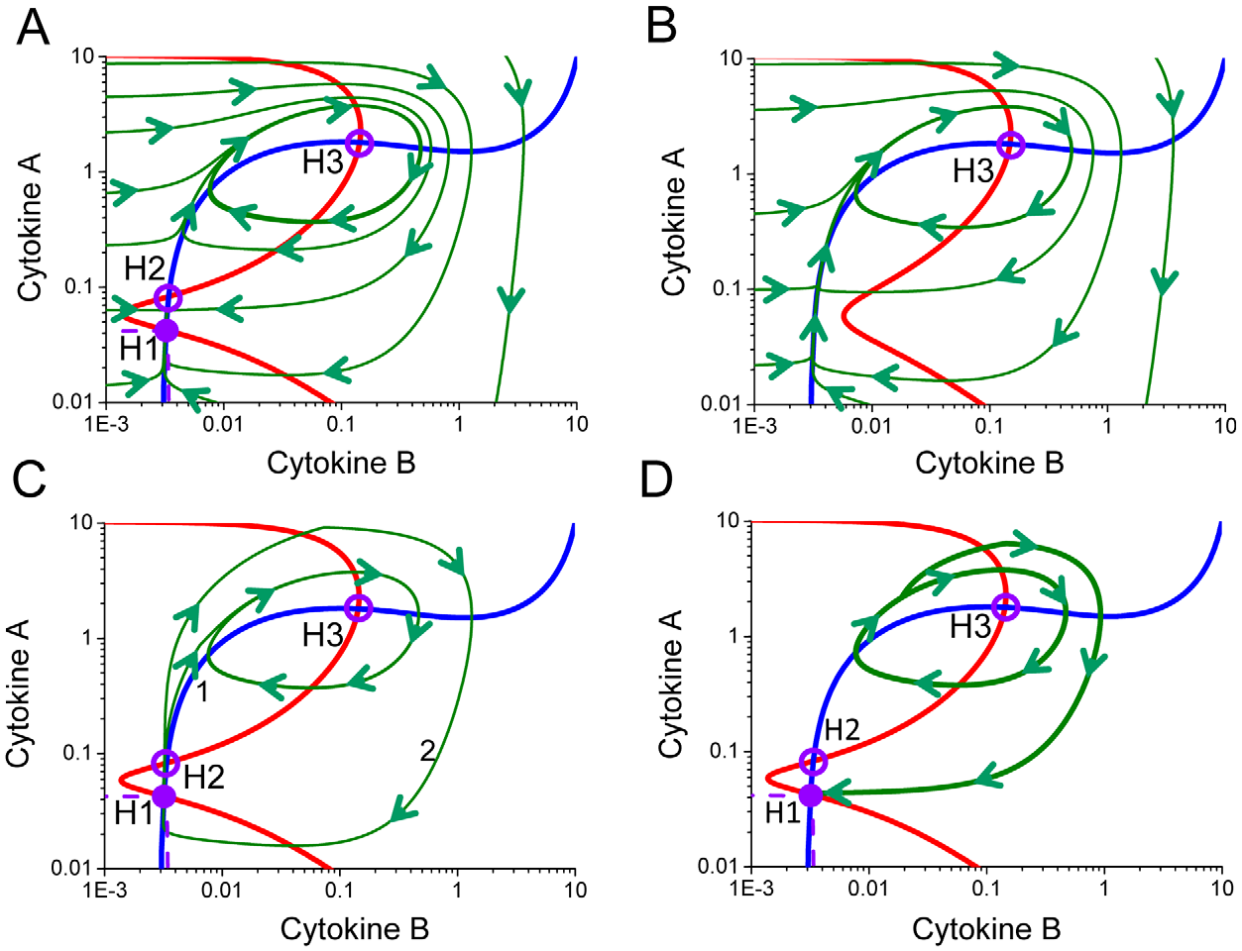
Oscillatory cytokine concentration dynamics. Internal or external factors can alter the cytokine production profiles and thereby modify the immune cell interaction parameters via feedback loops. Such modification can lead not only to the shift or appearance of new levels of homeostasis, but also to the loss of homeostatic stability with the appearance of limit cycles. A. The nullcline diagram shows the multiple homeostasis solutions as indicated by the intersections of the red and blue cytokine dose-response curves. The filled circle represents a stable solution, whereas the hollow circles demonstrate unstable solutions. One of the unstable solutions forms a limit cycle which represents the possibility for cytokine concentrations to oscillate. The green lines show how the system converges either into the stable homeostatic point or stable oscillations from any other combination of non-homeostatic cytokine concentrations. B. The nullcline diagram describes the case of one unstable solution that forms a limit cycle. C. External perturbations of variable amplitude can shift the system from the stable low cytokine concentration state into the mode of stable oscillations in the higher concentration range, as indicated by trajectory 1. Interestingly, higher amplitude perturbations, applied externally, cause the interdependent cell populations generate large spikes and followed by return to the homeostasis point bypassing the oscillatory mode (trajectory 2). D. An external impulse of small magnitude applied during the oscillatory regime is able to return the system into the basal level of homeostasis.

Under certain combination of parameters (Table 1), a stable solution H3 (Figure 6A) can become unstable (Figure 7A), and form a limit cycle that represents simultaneous oscillatory alterations of both cytokines. Stable oscillations of cytokine concentrations cause unbalanced proliferation and differentiation of keratinocytes, the main cell type constituting dermis and epidermis, and are thus pathologic for skin. At the same time, the oscillatory type of pathology is different from the cytokine-trigger mode described in the previous section. Trigger-like inflammation causes clearly defined areas of lesion, whereas oscillations are more likely to cause a phenotype with gradual transition between inflamed and non-inflamed areas of skin.

Variation of cytokine oscillation-driven pathology is shown on Figure 7B. The chosen combination of model parameters allows only one unstable solution H3 with the limit cycle in the area of high cytokine concentrations. The absence of stable homeostatic solutions leads to the most severe disease phenotypes, which are least susceptible to potential treatment.

**Figure 8:**
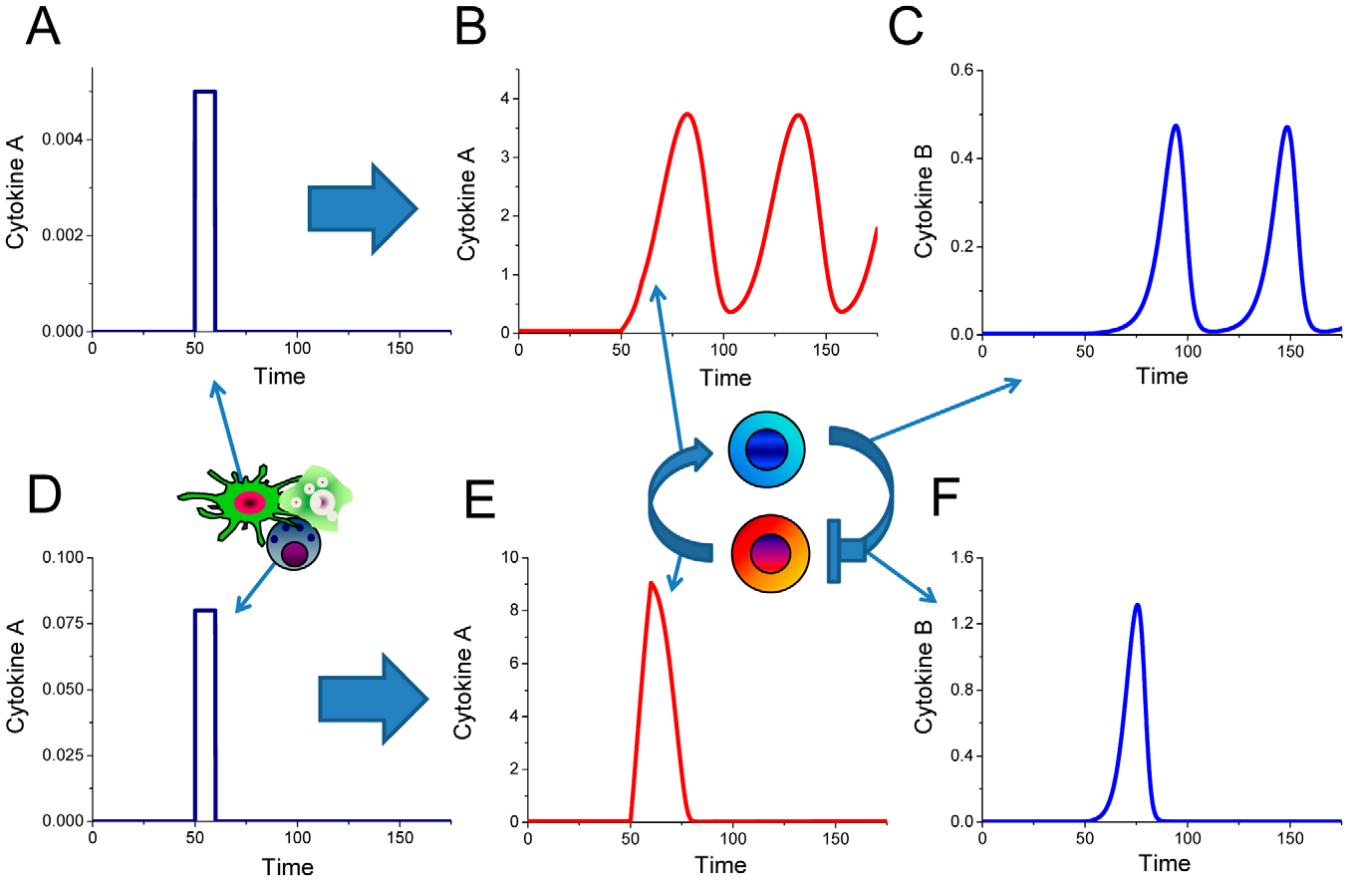
Temporal evolution of cytokine concentrations in response to applied perturbation. Variable magnitude impulses of cytokine A (A) and (D) applied to the interacting cell populations can shift the cells from the basal homeostasis point into the mode of stable oscillations (B) and (C) or generate a large spike and return into the homeostasis (E) and (F). The larger perturbation (D) causes the immune cell population system to generate a single impulse instead of undergoing stable oscillations. In both cases the magnitude of the perturbation is significantly smaller compare to the response generated by the interacting immune cells.

**Figure 9:**
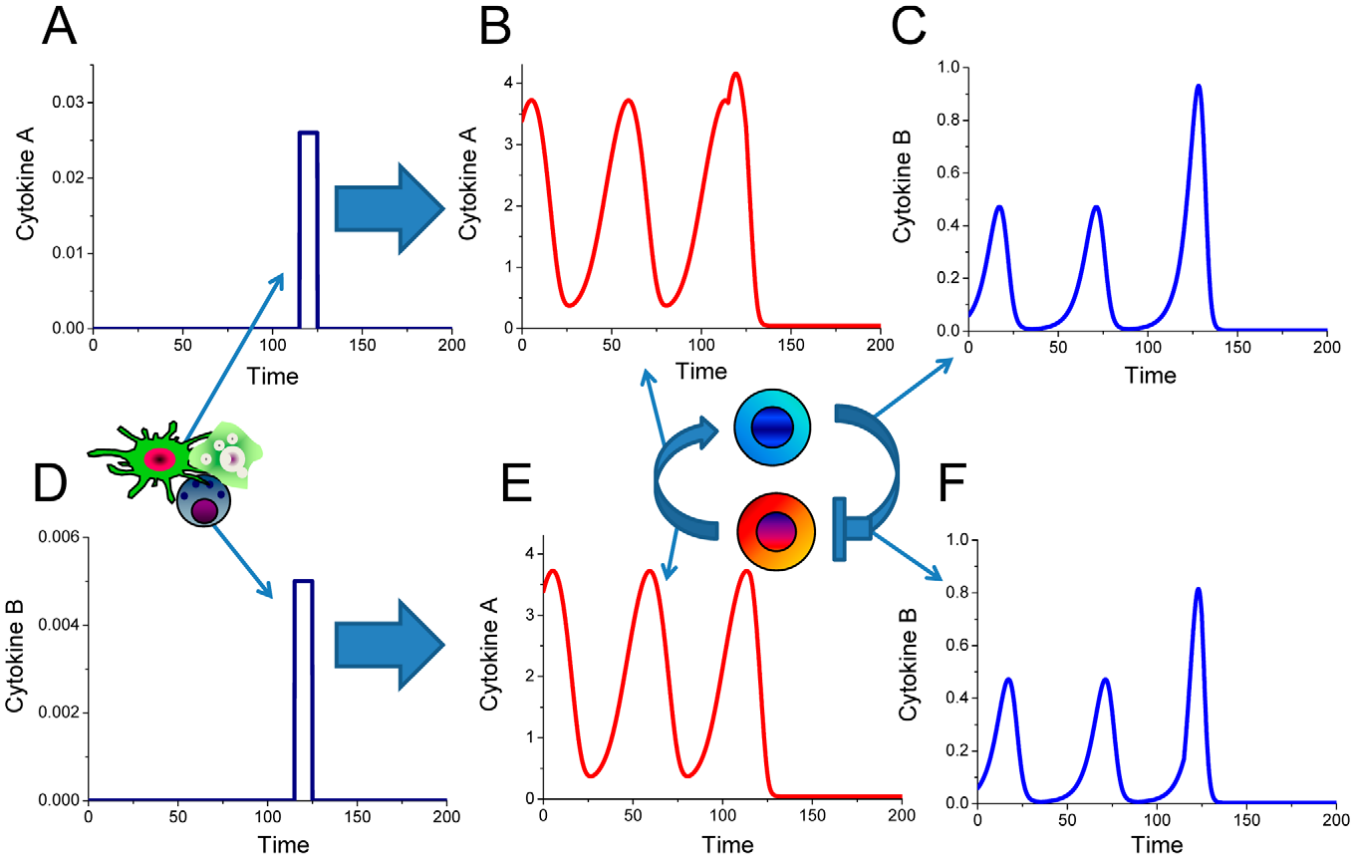
Model predictions for the transition from the oscillatory regime into the homeostatic level. Small external impulses of cytokines A (A) and cytokine B (D) applied for a temporal period of time can switch the immune system from generating stable oscillations back into the level of homeostasis. The dynamics of cytokine concentrations during the transition from the oscillatory mode back into homeostasis after the cytokine A and cytokine B perturbations are shown on (B–C) and (E–F), respectively.

In order to analyze the dynamic properties of immune cell interactions in relation to the type of pathology (Figure 7A) when cells either maintain the stable homeostasis or experience stable oscillations, we studied how the system responds to the applications of external cytokine concentrations. The present model predicts that the external cytokine A application can either switch the system from the homeostasis H1 to oscillatory mode around unstable solution H3 (trajectory 1 on Figure 7C) or generate an impulse and the system returns to homeostasis H1 (trajectory 2 on Figure 7C) as in the case of healthy immune reaction. The difference between healthy and pathologic responses is due to the amplitude of applied external perturbation of cytokine A (Figure 8). Small impulses shift the system out of homeostasis H1 into the oscillatory mode. The spike of higher amplitude leads to generation of a sizable response, before returning to homeostasis level H1. Our model predicts that small perturbations of either cytokine A or cytokine B is sufficient returning the system from oscillatory mode to the normal level of homeostasis (Figure 7D and Figure 9).

## Discussion

We propose a new systems biology model that captures crucial properties of immune cell interactions and predicts the conditions under which normal and pathological inflammatory responses are elicited. The model integrates individual characteristics of immune cell populations and allows the definition of homeostasis as specific cytokine concentrations estimated by the intersection of the immune cell population cytokine dose-response curves. The model predictions provide novel insights into the mechanism of elevated levels of inflammatory cytokines in disease [2], [15], [43]. While it is well known that (i) genetic variants change the susceptibility to disease [44] and (ii) the same disease phenotype can be elicited by different types of inflammation [15], the relationship between genetic variants and pathologic inflammation remains unclear. The present study reports a generic framework to explain why and how small alterations to cytokine production profiles (arising from genetic variants which can be different across cases and not always statistically significant) leads to the modification of feedback loop interactions between immune cells and the appearance of pathologic inflammatory levels.

This study suggests that cytokine concentrations can deviate from homeostatic levels even in the absence of any pathology, as long as such deviations are temporal and always return to homeostatic level in equilibrium. Normal immune response initiates temporal increase of key cytokines concentrations for a time span sufficient to execute the effector system and eliminate the cause of inflammatory reaction. Pathology occurs if the inflammatory response is not temporal and cytokine concentrations fail to return to the original levels. According to the model predictions, homeostatic cytokine concentrations can only be estimated from the interactions of interdependent cell populations. Homeostasis is therefore a systems effect and occurs at the crossing of the dose-dependent cytokine productions curves from at least two immune cell populations (Figures 4D and 4E). The analysis of model properties allows unravelling of mechanisms that cause stable chronic inflammation. According to the model, normal immune system can be described as a system with one stable homeostatic level defined by the cytokine feedback loop parameters of immune cell interactions. External perturbations applied to the healthy immune system induce a temporal cytokine concentration increase, followed by a return to the stable homeostasis (Figure 5).

Alterations in the feedback loop parameters [18]–[23], [45] can turn the immune system pathologic by inducing bistable behavior with discrete steady-states or loss of stability in homeostasis. The present study follows earlier modeling analyses of different types of inflammation [28]–[29], [32], [42], [46]–[53]. Similarly to previous studies, the framework reported here predicts that inflammatory response is a highly dynamic process that can be represented mathematically by incorporating experimentally derived feedback loop interactions between immune cell interactions. The presented model proposes new generic principles that can distinguish healthy and pathologic inflammation. Moreover, it offers a rational foundation to establish the relationship between causative genetic variants, alterations in the cytokine production profiles and modifications in the feedback loop interactions between immune cells, ultimately leading to the appearance of inflammatory pathology. The model also possesses a predictive capacity to distinguish between different types of inflammation that can arise from the same immune system. Overall, the application of systems modeling theory to simulate the immune cell regulation effects in psoriasis through altered properties of feedback loops can outline the key factors that distinguish normal immune system response from pathology.

The quantitative model for immune cell interactions in this study offers a mechanistic distinction between healthy inflammatory reaction and pathological inflammation. Internal and environmental factors can alter cellular interactions in the form of modified cytokine production curves. In order to investigate how such alterations can translate into various pathologies, the derived model was subjected to exhaustive evaluation of the underlying parameters of cytokine production and degradation rates without any modifications in the model structure. Such an assumption reflects the physiological situation where the interacting immune cell population pairs remain the same, but the parameters of the interactions can vary due to genetic mutations. The model predicts that the autoimmune mediated pathology occurs in those cases where the modified feedback interactions between immune cells lead to the appearance of additional levels of homeostasis (Figure 6). As a result, the immune system can start operating as a cytokine trigger and maintains either low or high cytokine concentrations levels. A different type of pathology can occur when the alterations of the cytokine-mediated feedback interactions between immune cells lead to the loss of stability of the homeostatic level. Variability in the interactions between immune cell populations can result in the appearance of oscillations (Figures 7, 8 and 9). The model therefore predicts that the same immune cell populations are not capable of mediating a normal immune reaction or operate as a biological trigger, instead, the immune system undergoes periodic temporal alterations. Stable oscillations of cytokine levels are also pathologic. The oscillations-based type of pathology is different from the trigger-type immune system pathology.

The healthy homeostatic and pathologic model predictions have been obtained through exhaustive screening of possible parameter values. The summary of representative sets of parameters chosen approximately in the middle range of the corresponding dynamic behavior is found in Table 1. While the listed parameter values may not be the only possible combinations of healthy and pathologic immune cell interactions for the described scenarios, they cover all possible types of dynamic behavior that the present model can achieve. One can choose different combinations of constants for the model so that it would oscillate or operate as a trigger, however there are no possible combinations of parameters where three or more stable homeostatic levels can exist, as it has been shown for example in multisite phosphorylation systems [54].

The combinations of parameter values are closely related to the model application on actual cytokines and, as noted earlier, two potential candidates for the proposed model are IL-17 and IL-23. Other cytokines that have been shown to be essential in skin inflammation include IL-22, oncostatin M, TNF-α, IL-1α [55], IL-6, IL-12, interferon-α and interferon-γ [15]. The difficulty of analyzing real cytokines rather than the immune cell interactions via hypothetical cytokines can be attributed to the fact that the majority of experimental investigations report static comparisons between experimental groups without considering either dose-dependent curves or dynamic information. While such comparisons are important, this model suggests that they may be insufficient for deeper understanding of mechanisms in inflammation. Further experimental investigations directed toward the dose-dependent cytokine production profiles would be required for estimation of the model parameters. It is essential to note that parameter values will be different in individual immune cell population pairs in a given tissue and that pathologic parameter alterations will depend on the combination of the causative genetic mutations found in specific cytokines.

**Figure 10:**
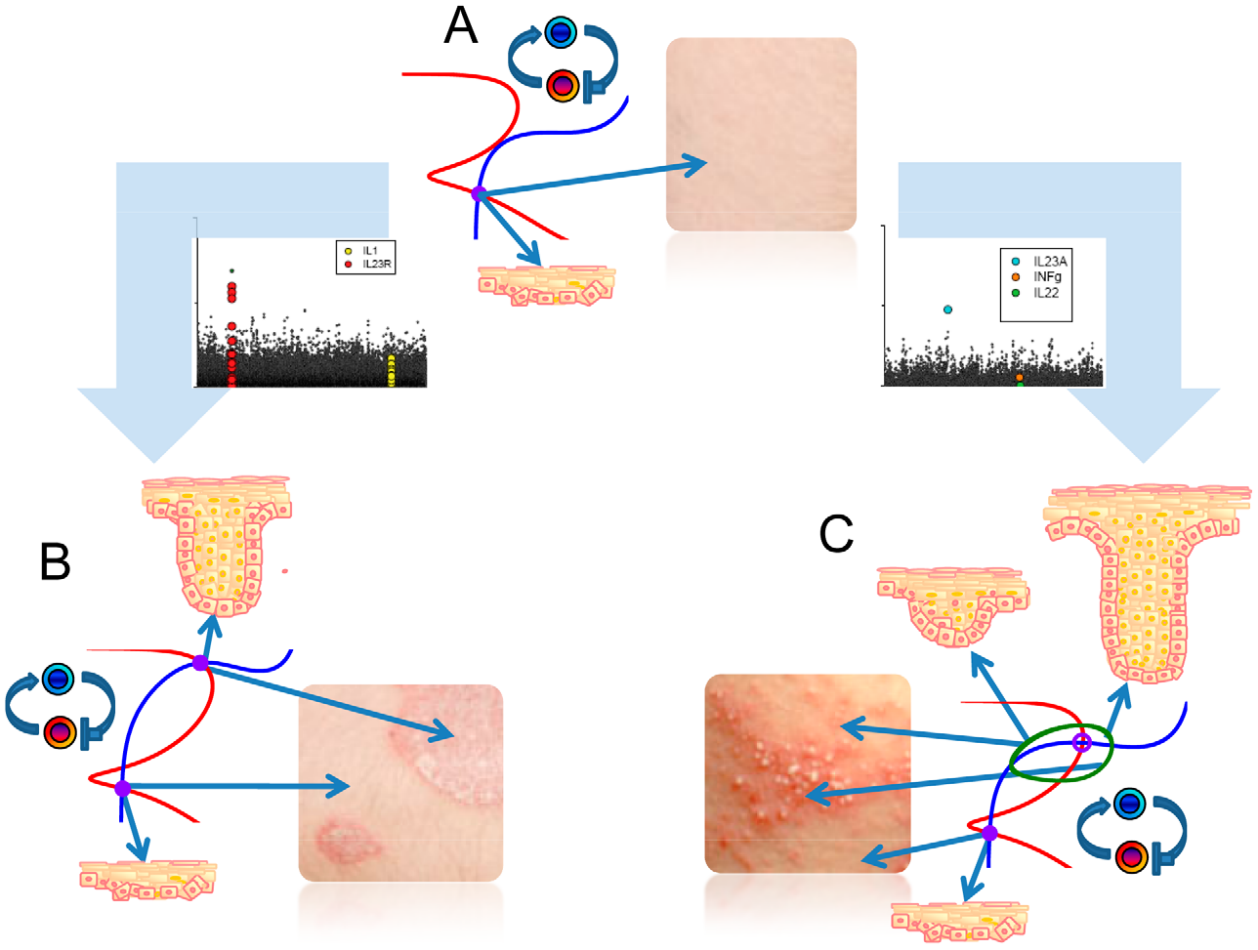
Systems biology description of inflammation in human skin. (A) Under normal conditions the homeostasis (defined by the dose-dependent cytokine production curve intersection) is reached at one steady-state point at low cytokine concentration levels. Combinations of SNPs and modified cytokine expression levels observed in disease can cause more than one stable (B) or unstable (C) homeostasis. In case of additional stable cytokine level (B), the interacting immune cell populations represent a trigger that can switch and remain in the state of either low or high cytokine concentration levels. When the combination of genetic alterations causes an additional homeostasis point which is unstable with a limit cycle, the cytokine levels can oscillate both locally and spatiotemporally. In such case, the inflammatory cytokines are more likely to be distributed more unevenly across the site of inflammation causing a skin inflammation phenotype of heterogeneous nature (C).

Figure 10 summarizes the model-based description of normal and pathologic immune system performance in human skin. Under normal conditions, cytokine production mediated interactions between immune cells lead to one stable homeostatic level in tissue (Figure 10A). Combinations of the internal and external factors can change the interactions between immune cells, in such a way that additional stable or unstable homeostasis levels appear. Chronically increased cytokine concentrations are more likely to be observed in clearly defined inflamed lesions (Figure 10B). In those cases, the immune system is able to switch and remain at the elevated cytokine concentration state. Oscillating cytokine concentrations are likely to cause a different inflammation phenotype with diffused borders between inflamed and non-inflamed regions (Figure 10C). The model predicts that the immune system's ability to mediate either normal or pathologic inflammatory responses is a systemic effect which emerges from the imbalance of immune cell interactions, rather than an attributed feature of a favorite cell population or a genetic polymorphism.

According to the proposed model, pathology occurs as a result of one or a combination of SNPs in cytokine or any other genes with the net effect of altered type of homeostatic level via the modification of parameters in the feedback loop interactions between immune cells. The description of the homeostatic mechanism from the systems perspective explains why SNPs in some cytokines (e.g. in IL-22), can have very low statistical association with psoriasis, but can contribute to pathology in a number of cases (Figure 2). The proposed mechanistic description of inflammation suggests that different combinations of SNPs (some or all of which can have very low association with the disease) can cause similar cytokine production curve alterations.

The proposed quantitative model for immune system explains how normal and pathologic inflammatory immune reactions can be mediated by the same immune cell populations. Current research in immuno-genetics mainly focuses on the search of polymorphisms highlighting candidate genes responsible for pathological inflammation. This work proposes that the altered feedback loop parameters (potentially arising from genetic polymorphisms) in the interactions between immune cell populations participate in the maintenance of inflamed lesions. The system model predictions for the possible coexistence of multiple homeostatic levels explains how inflammatory disease affected individuals can simultaneously have both non-inflamed and inflamed areas of skin while carrying the same genotype with disease-associated SNPs. The proposed approach, therefore, offers a mechanistic explanation for why “causative” SNPs mediate inflammatory lesions at some regions of skin while they do not do so at others.

### Therapeutic applications of systems model for immune cell interactions

The systems model described in this work is relatively generic and applicable to analysis of a range of inflammatory conditions. The mathematical model allows the prediction of mechanisms in inflammatory disease and the formulation of requirements for therapeutic interventions. The model-guided screening of therapeutic agents can be performed on the bases of eliminating the second possible level of stable or unstable homeostasis or lowering existing cytokine concentrations.

**Figure 11:**
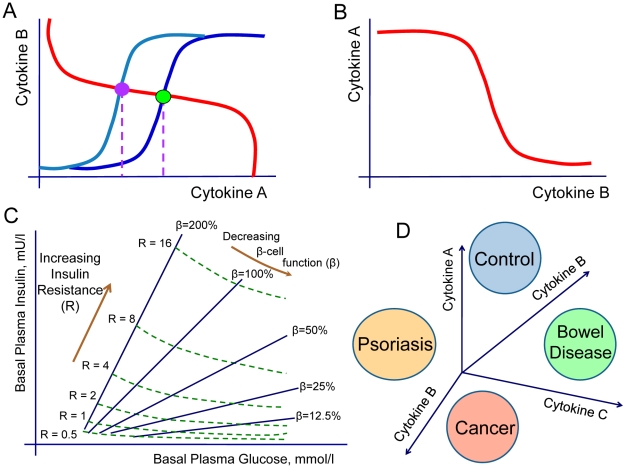
Therapeutic applications of systems model for immune cell interactions. The systems model for cytokine-mediated immune cell population interactions offers new strategies for development of pharmaceutical interventions. A. The altered cytokine production profiles lead to the modification of feedback parameters between immune cell populations. Modified feedback changes the level of steady-state homeostatic for individual cytokines. The lower and higher homeostatic concentrations for the cytokine A, indicated by violet and green circles, take place for two dose-dependent cytokine profiles from normal and pathologic immune cell populations. In this case, potential therapeutic strategies may focus on identification such compounds that will rescue the original cytokine production profile. B. The interdependence of cytokines via cell populations suggests new strategies for indirect therapeutic interventions by cytokine injections. In this example, injections of cytokine B are likely to decrease the levels of cytokine A. C. The graph, adopted from [26], is an example of the computational homeostatic model that model determines the steady-state basal plasma glucose and insulin concentrations by their interaction in a feedback loop. Comparison of a patient's fasting values with the model's predictions allows a quantitative assessment of the contributions of insulin resistance and deficient β-cell function in type II diabetes. D. In analogy with the homeostatic model assessment in type II diabetes [26] (C), the proposed model for immune cell interactions contains predictive potential for quantitative determination of inflammation-related pathologies.

The model describes different pathology phenotypes which are due to the appearance of additional stable or unstable levels of homeostasis, the loss of stability of the basal level of homeostasis or due to the shift of homeostasis to the levels of higher cytokine concentrations. The last case is probably the most frequent and “simple” scenario of inflammatory pathology that occurs when the cytokine production curves intersect at higher cytokine concentration levels. For example, different homeostatic concentrations of a cytokine A shown on Figure 11A occur as a result of altered cytokine production profiles by immune cell populations. According to the proposed methodology, the search for pharmaceutical interventions can be based on identifications of direct or indirect way to restore the original dose-response profile of immune cell population. The interdependence between cytokines via an immune cell population can be utilized by indirect target identification strategies for novel interventions, by using already available therapeutic agents. One possibility is the injections of a cytokine B know to reduce the levels of a different cytokine A (Figure 11B).

**Figure 12:**
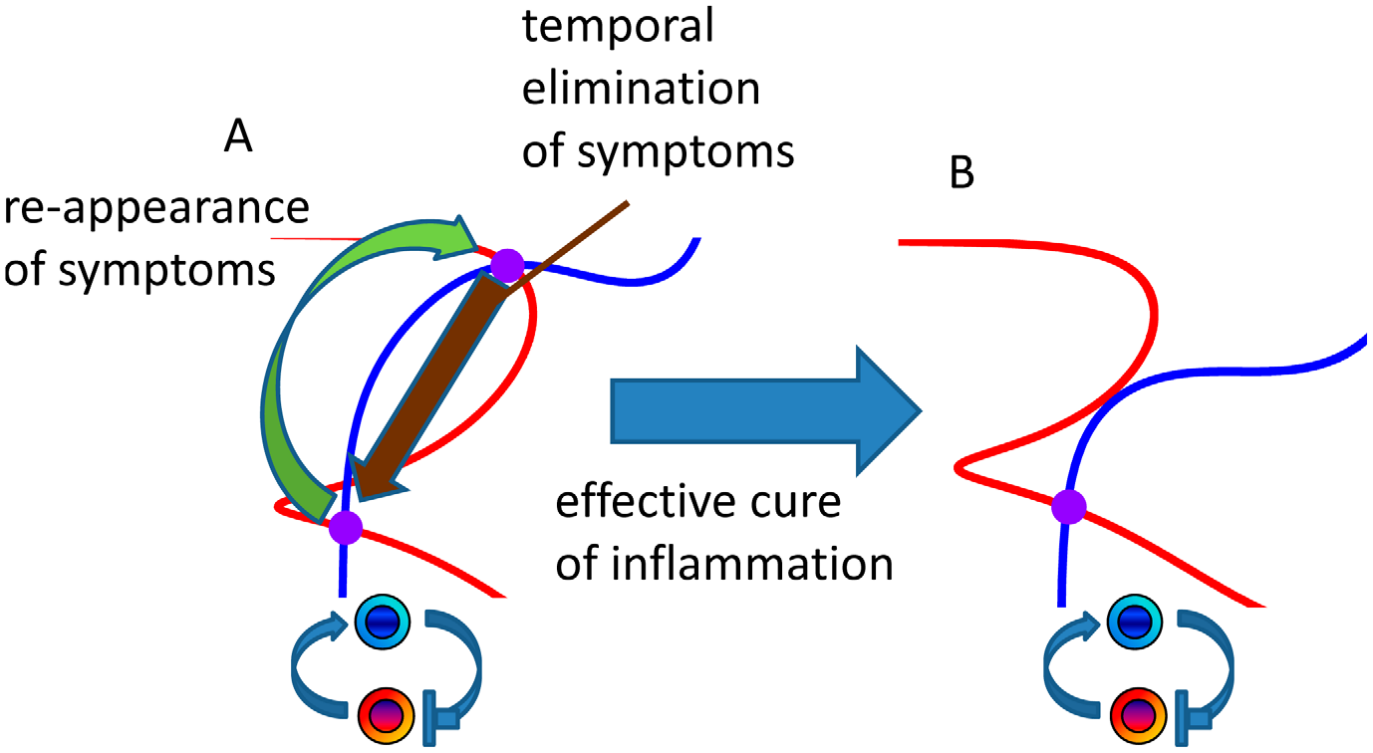
Systems interpretation of pharmacological agent effects on inflamed tissue. The majority of currently available pharmacological agents allow temporal elimination of inflammatory symptoms. In the context of the proposed systems model, this effect can be considered as a switch from the inflamed to perilesional steady-state (A). While such compounds or antibodies offer temporal relieve from inflammatory symptoms, they do not represent effective means of cure. The new pharmacological agents can be developed and selected on the action that leads to the disappearance of the additional inflammatory level (B).

The proposed mathematical approach offers new exciting therapeutic opportunities for various inflammatory conditions. One interesting example where the ideas proposed in this study have already been utilized in a similar fashion is the type II diabetes. There are two ways of estimating insulin resistance in diabetes: the glucose clamp test [56] and the homeostasis model assessment [26]. The homeostasis model assessment system seeks values of resistance to the hypoglycemic effect of insulin and β-cell function from the measures of plasma insulin versus glucose, in comparison with a standard group of healthy young adults. The homeostasis model assessment approach takes into the consideration the interactions between glucose and insulin via the specialized cell populations (Figure 11C) and thereby increases diagnostic power in diabetes [26]. We propose that the model for quantitative inflammation assessment will offer advanced diagnostic tools for inflammatory conditions, as opposed to diagnostic methods based on readouts of a single biomarker (Figure 11D). The model suggests the necessary criterion for the properties of required treatment. A large number of currently available pharmaceutical agents offer a temporal relieve from the inflammation induced symptoms. In the context of systems representation of the disease, the drug action can be viewed as a temporal switch from higher to lower cytokine concentration steady state (Figure 12A). Any physiological alterations are likely to switch the system back into the level of pathological inflammation. The major criterion for the new treatments would require them to eliminate the second pathologic steady-state level (Figure 12B).

### Systems model suggests new avenues for data interpretation

**Figure 13:**
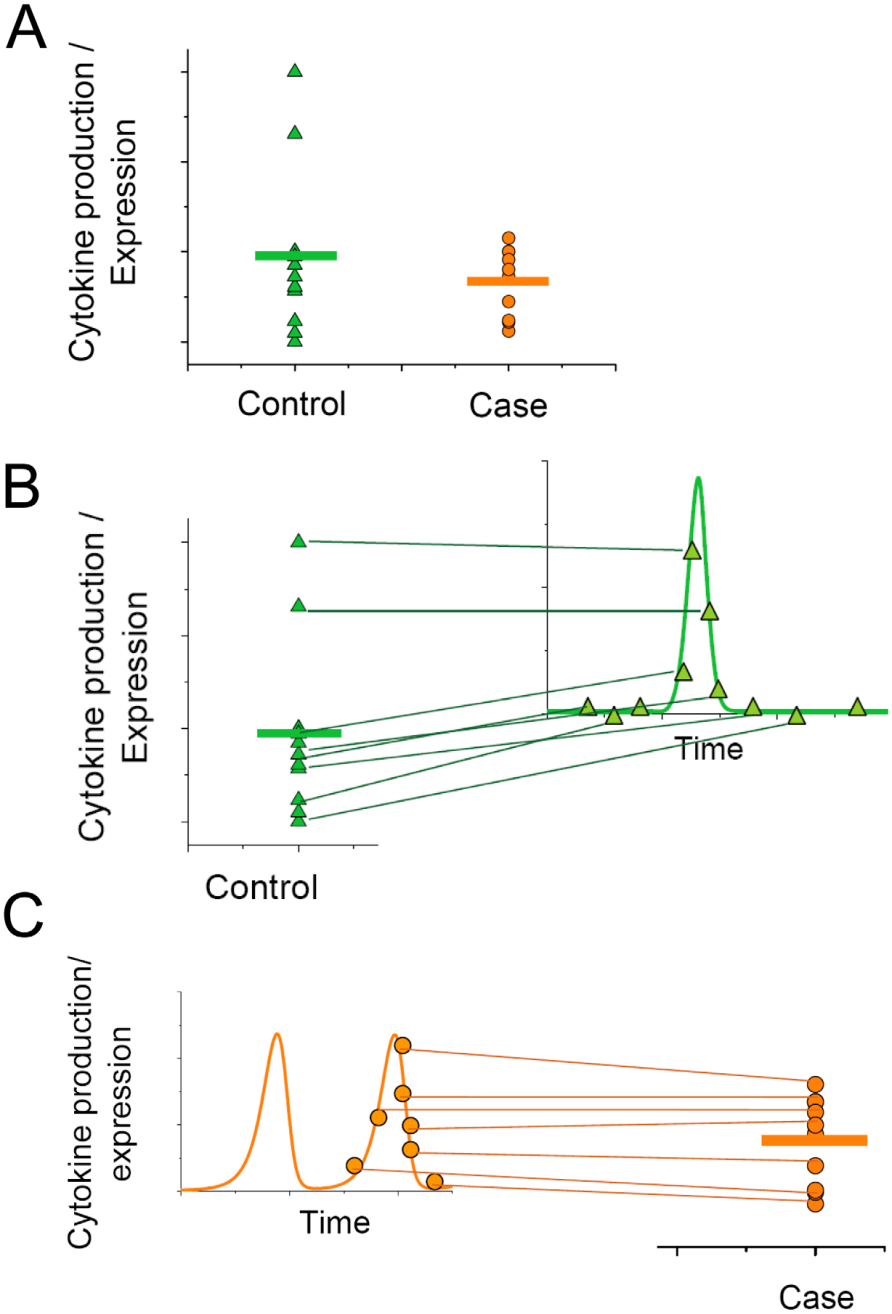
The dynamics dimension for the data interpretation. A. The comparison of cytokine production levels between may not provide statistically significant differences between health and pathology. More importantly, the widely used approach does not offer insights into the mechanism of the observed differences. B. The representation of an immune system as a system of interdependent cells interconnected by the cytokine production and degradation mechanisms provides new possibilities of data interpretation. Experimentally observed readout variability can be interpreted as time course points during an immune response. C. The time course perspective shows the possibility of oscillatory cytokine dynamics.

Systems modeling of inflammatory responses initiated by interdependent immune cell populations can offer new avenues for inflammatory disease-associated data interpretation. In a vast number of cases, the comparison between cytokine production or expression levels is performed statically, by calculating the medians between readouts obtained from cases and controls (Figure 13A). Such representation does not capture the regulatory alterations in cytokine expression or production during either normal or pathological events. As a result, there can be a significant variability in the experimental readouts. However, if data are viewed from a systems perspective, the possibility of dynamic alterations would explain the observed variability in both cases (Figure 13B) and controls (Figure 13C). The combination of the immune cell interactions in the form of a dynamic model with the measured cytokine production or expression levels in heath and disease can offer more explanation for the experimentally observed data points.

### Future perspective

One of the major difficulties in research of inflammatory pathologies is the lack of unambiguous definition of disease. The systems model suggests that inflammatory skin disease is unlikely to be mediated by one gene or by a specific cell population. Instead, local inflammation of the immune system in the skin arises from systems-level effects emerging from the interactions between cell populations via cytokines, chemokines and cell surface expressed ligands. Interdependent cytokine production by cell populations creates a network of immune cells with a number of emergent properties such as integration of signals across the immune system, generation of distinct outputs depending on combinations of internal and external conditions. Of particular interest is the immune system ability to form discreet steady-states and switch between them. This study analyzes the effects arising from the interaction of two cell populations only. While the mathematical model covers the range of large number of possibilities, one needs to acknowledge that more sophisticated effects can arise from larger number of interacting cell populations via cytokines. Current study does not include a specific dose-dependent or time course data for cytokine dynamics. While inflammation-associated pathologies are likely to develop according the described principles, a specific subset of cytokines and immune cell populations is needed to be identified for each specific inflammatory condition.

## Materials and Methods

### Genome-wide association study analysis

The high quality genotypes for 438,670 markers of the 1359 psoriasis cases and 1400 controls from the genome-wide association scan performed by the Collaborative Association Study of Psoriasis [17], were used for association analysis. The dataset used for the analyses described in this manuscript were obtained from the database of Genotypes and Phenotypes (dbGaP) found at http://www.ncbi.nlm.nih.gov/gap through dbGaP accession number phs000019.v1.p1. Single marker case – control association analysis was performed by executing the –*assoc* option of the PLINK package (v1.06) developed by Shaun Purcell (http://pngu.mgh.harvard.edu/purcell/plink/) [57]. This option calculates the statistical significance as measured in odd ratios, *P* or χ^2^ values of the minor allele frequency differences between psoriatic cases and healthy controls.

### Inflammatory cytokine significance evaluation in the whole genome-wide context

Genome-wide association of each SNP is showed in a Manhattan plot as the −log_10_ (*P*) dependence on the genomic location using the coordinates of the NCBI Build 36.1 (March 2006). The association of the SNPs located within the 2 Mbp window centered at the selected inflammatory cytokines is shown in color for individual cytokines (Figure 2C).

### Integrative systems biology model for immune cell interactions

Figure 4 provides a schematic framework of the two interacting cell populations. We investigate the interactions between immune cell population and the interaction-dependent properties of the immune system in homeostasis through a mathematical model that captures the extracellular cytokine concentrations. All possibilities of immune cell interactions are cdescribed in Supplementary Text S1. Given that cytokine production by immune cell populations can be represented as a function of another cytokine in a dose-dependent manner, inflammation can be defined quantitatively by considering cytokines as interdependent variables, where the specific inter-dependence of cytokines can be established experimentally through studying immune cell populations. The interdependence of cytokines A and B can be represented by a system of coupled ordinary differential equations:
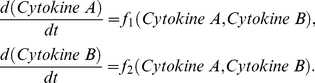
(1)The same principle can be applied to larger numbers of cytokines and chemokines produced by immune cell populations:
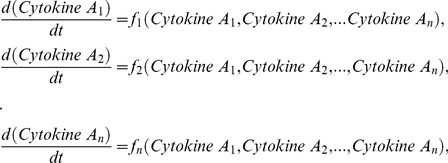
(2)where *n* is the total number of considered cytokines.

In order to elucidate what distinguishes normal and pathological immune system performance, two cytokines interconnected via dose-dependent effects of corresponding cell populations are considered. Effects that occur in the multidimensional space of cell interactions via cytokines can be projected to two dimensions and we show below that alterations in an immune sub-system with two interacting cell immune cell populations have the potential to describe several different inflammatory phenotypes. We develop a systems model for cytokine, chemokine and surface ligand-mediated immune cell interactions that can unravel the mechanism of inflammation and provide mechanistic explanation for the inflammation in human skin. The model contains two cell populations interconnected via activatory and inhibitory cytokine production. The dose-dependent cytokine production is complemented by cytokine removal via diffusion, cleavage by metalloproteases and trapping mechanisms.

In the most general case, the speed of cytokine concentration 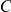 dynamics in tissue can be represented as follows:
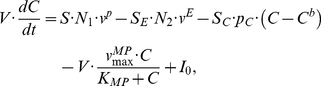
(3)where 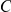 is the cytokine concentration, 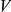 is an elementary tissue volume, 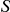 is a surface area of a cell that produces a cytokine, 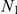 is the number of cells that produce a cytokine in volume 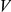, 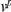 is the rate of cytokine production 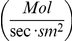, 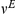 is the rate of cytokine uptake via endocytosis, 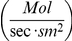, 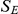 is a surface area of cells that express the cytokine receptor, 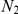 is the number of cells capable of endocytosis of the cytokine receptor in volume 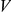, 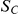 is the capillary surface area in the volume *V*, 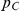 is the capillary permeability to the cytokine, 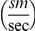, 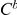 is the cytokine concentration in blood, 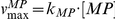 is the maximum cytokine degradation rate by proteases 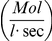, 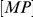 is the concentration of proteases, 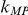 is the rate constant 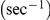, 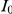 is the basal cytokine secretion rate by an immune cell population 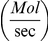. 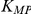 is the Michaelis constant.

In order to develop the mathematical model capturing the interactions in immune cells via cytokines we defined a number of following immune cell subpopulation groups according to the classification shown on Figure 4: i) cells produce cytokine B in a dose-dependent manner from cytokine A (Figure 4B), ii) the production of cytokine A is inhibited by a cytokine B (Figure 4C). One can also consider a variety of other cases of the bell-shape or reverse bell-shape dependence on cytokine concentration or even more complex cases. We analyze the cytokine system properties under the framework of outlined assumptions and any specific cytokine-dependent cytokine production profiles can be developed as an extension of the model described below.

The rate of cytokine A production by a cell population when it interacts with another population that produces inhibitory cytokine B is given by:
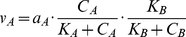
(4)where 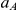 is a normalization coefficient, 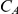 is the cytokine A concentration, 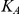 is the dissociation constant for the cytokine A interaction with the cytokine A receptor, 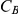 is the cytokine B concentration, 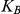 is the dissociation constant for the cytokine B interaction with the cytokine B receptor.

The rate of the cytokine B production by a cell population when it interacts with another population that produces activatory cytokine A is given by:
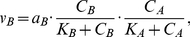
(5)where 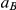 (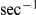) is a normalization coefficient.

Cytokine production by a given cell populations can be modulated by several activatory or inhibitory cytokines. In this general case the cytokine production is given by:
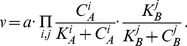
(6)where 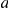 is a normalization coefficient, 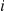 and 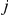 are the numbers of activatory and inhibitory cytokines, respectively. 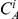 is the concentration of the 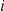
th activatory cytokine and 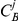 is the concentration of the 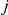
th inhibitory cytokine. 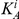 is the dissociation constant for the cytokine 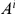 interaction with the cytokine 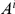 receptor. 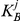 is the dissociation constant for the cytokine 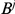 interaction with the cytokine 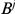 receptor.

Cytokine production is complemented by mechanisms of cytokine elimination. Various routes of cytokine removal from extracellular space include cleavage by metalloproteases, diffusion, cytokine trapping, binding to the cytokine receptor and uptake. Cytokine removal by diffusion and cleavage by metalloproteases are nonspecific and do not play an active role in the regulation of the extracellular cytokine concentrations, whereas the cytokine binding to the receptor followed by either release or uptake can have significant implications on the cytokine concentration dynamics. Thus, we next develop governing equations for the cytokine-cytokine receptor interactions.

Cytokine binding to the receptor initiates intracellular signaling events. Under the conditions of dynamic equilibrium, in the absence of endocytosis, the number of cytokines bound to the soluble receptors would equal to the number of cytokines released. However, due to the cytokine-cytokine receptor complexes uptake certain amount of cytokine is internalized via endocytosis mechanism and degraded. The cytokine uptake decreases the cytokine concentration in the extracellular space in the cytokine concentration-dependent manner. The rate of cytokine uptake 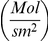 by a cell population is proportional to the number of receptors bound to the cytokine, multiplied by the total number of receptors on the cumulative cell surface:
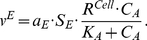
(7)where 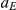 (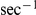) is a normalization coefficient, 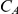 is the concentration of cytokine A, 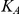 is the dissociation constant for the cytokine 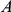 interaction with the cytokine 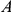 receptor. 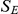 is the cell surface area, 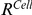 is the number of receptors expressed on a cell surface.

The total number of receptors can be divided into two fractions: receptors that are present on the surface and the subpopulation in the vesicles after the uptake event took place. In steady-state, the rate of receptor synthesis equals to the rate of receptor degradation by proteosomes; these rates are not considered in the present analysis. The conservation law applied to the two receptor populations at any given time point is given by:
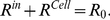
(8)where 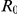 is the total number of receptors to a specific cytokine 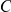, 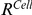 is the number of receptors on the cell surface, 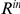 is the number of internalized receptors.

The dynamics of the receptors present on the cell surface is given by:
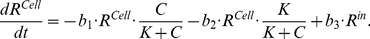
(9)where 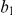 and 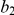 are the coefficients that describe the rate of cytokine bound and cytokine free receptor internalization, respectively. 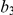 reflects the rate of receptor recovery from proteosomes, 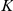 is the dissociation constant for the cytokine 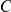 interaction with the cytokine 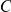 receptor, 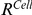 is the number of receptors on the cell surface, 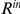 is the number of internalized receptors.

In the steady-state, the number of receptors on the cell surface as a function of extracellular cytokine concentration is given by:
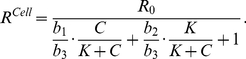
(10)The combination of equations (10) and (7) allows obtaining the rate of cytokine uptake as a function of cytokine concentration:
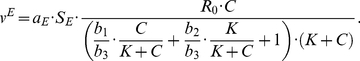
(11)The full model for the immune cell interactions is given by:
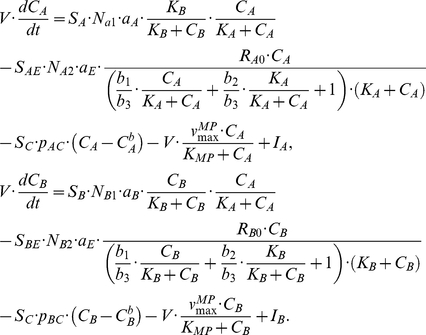
(12)


### Relationship between parameters in original and normalized model equations

The relationship between the parameters in the normalized system of differential equations with the original description for the cytokine production and uptake rates is thus given by:
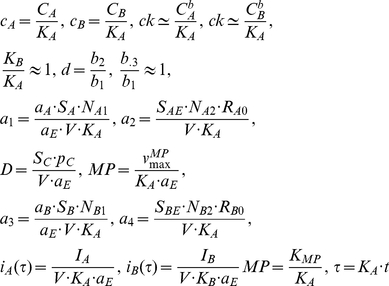
The final system of differential equations for two interacting cell populations which was solved numerically to generate all the results presented in the paper is thus given by:
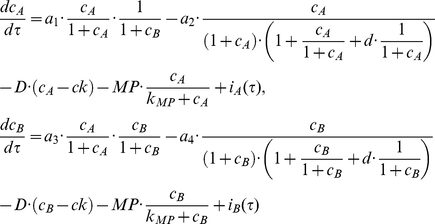
(13)All parameter values used in the above equations are given in Table 1.

## Supporting Information

Text S1 General principles of cytokine-dependent immune cell population interactions.
(1.09 MB PDF)
