# Supplementary material for: A Systems Model for Immune Cell Interactions Unravels the Mechanism of Inflammation in Human Skin
Source: PLoS Comput Biol. 2010 Dec 2;6(12):e1001024. doi: 10.1371/journal.pcbi.1001024 (PMC2996319; doi:10.1371/journal.pcbi.1001024)
Supplement: Text S1 — General principles of cytokine-dependent immune cell population interactions. (1.09 MB PDF) [file pcbi.1001024.s001.pdf]

SUPPLEMENTARY MATERIAL

This section provides background on the cytokine-mediated interactions between cells and possible therapeutical implications of the results. The concepts are communicated in sufficient details allowing an expert in the area to understand and apply the described principles to specific systems.

Immune cell population modulation by extracellular cytokine concentrations

From the range of the methods currently employed, such as GWAS, cytokine expression or production measurements, the alteration of cytokine production profile is probably the most physiologically relevant factor that reflects the modifications of the feedback interactions between the cells. Given the numerous examples of cytokine-mediated feedback control [1-7], we describe the general principles of cytokine-dependent immune cell population regulation in the context of human disease.

Regardless of whether a cytokine is soluble or expressed on the cell surface, the effect of cytokine A to an immune cell population that produces cytokine B can be classified as shown in Figure S1 schematically. A cell population with expressed receptors to cytokine A can produce cytokine B (Figure S1A) in the cytokine A dose-dependent manner. The dependence of cytokine B production on cytokine A in tissue is not due to single immune cell population, instead it is a cumulative effect of all cells that produce cytokine B with functionally expressed cytokine A receptor. Figure S1 summarizes the possible cytokine production profiles. Cytokine B concentration can increase with increasing extracellular cytokine A concentration, (cytokine A is *activatory*, Figure S1B). Cytokine B production can be at a maximum in the absence of cytokine A and then decrease as cytokine A levels increase, (cytokine A is *inhibitory*, Figure S1C) Cytokine A can have a bell-shaped or reverse bell shaped effect on cytokine B production in an immune cell population (Figures S1D and S1E respectively). In these cases, cytokine A acts as either *activatory* or *inhibitory* depending on the concentration range. Figure S1F shows the case when the cell population either does not

possess cytokine A receptors, or - in the presence of cytokine A receptors - intracellular signaling circuits do not mediate any effect on cytokine B production. While we only consider five possibilities of one cytokine effect to another mediated via a cell population, other more complex dose-dependent profiles can be represented as combinations of these cases.

FIGURE S1

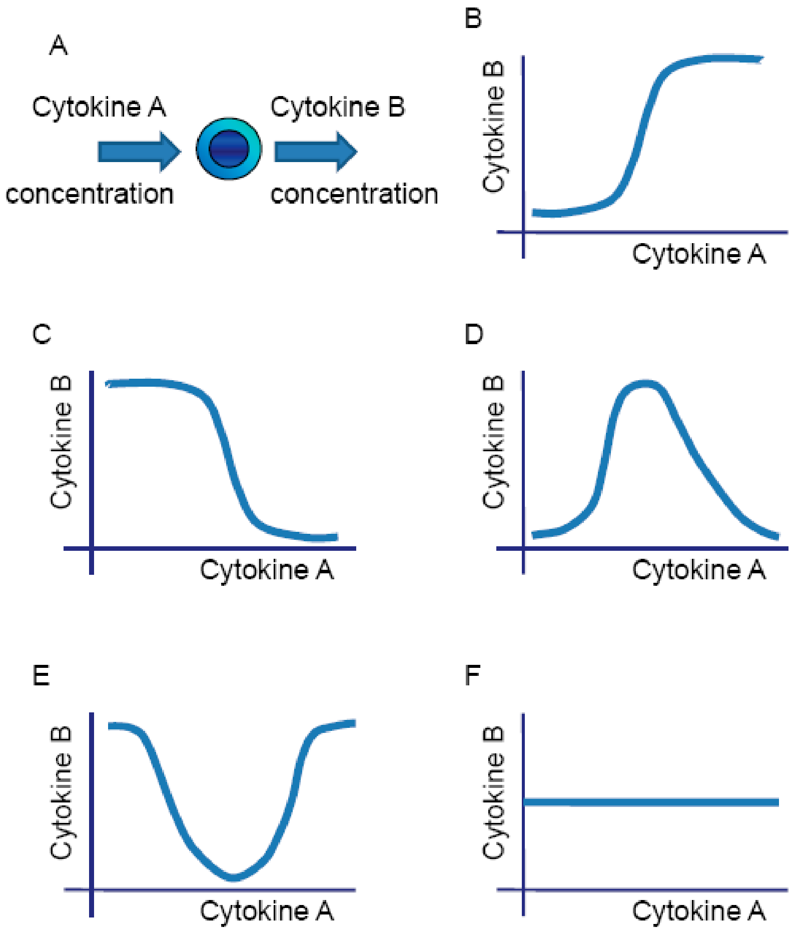

Figure S1. Summary of the possible dose-dependent cytokine production rates.
A. An immune cell population can produce cytokines or chemokines as a function of external cytokine and chemokine concentrations. In most cases it is possible to identify and measure the production of cytokine B as a function of external cytokine A concentration.

The range of possibilities for a cytokine B production dependence on cytokine A by immune cell populations include: dose-dependent increase (B) or decrease (C), bell- (D) or reverse bell-shaped (E) curves. Cases where a cell population produces a cytokine B with no dependence on cytokine A even in the presence of cytokine A receptors are represented by (F).

More complex cytokine production dose-dependent shapes can be generated by combinations of the (B-F) cases.

References

1. Darrasse-Jeze G, Deroubaix S, Mouquet H, Victora GD, Eisenreich T, et al. (2009) Feedback control of regulatory T cell homeostasis by dendritic cells in vivo. J Exp Med.
2. Gabrysova L, Nicolson KS, Streeter HB, Verhagen J, Sabatos-Peyton CA, et al. (2009) Negative feedback control of the autoimmune response through antigen-induced differentiation of IL-10-secreting Th1 cells. J Exp Med 206: 1755-1767.
3. Harper EG, Guo C, Rizzo H, Lillis JV, Kurtz SE, et al. (2009) Th17 Cytokines Stimulate CCL20 Expression in Keratinocytes In Vitro and In Vivo: Implications for Psoriasis Pathogenesis. J Invest Dermatol.
4. Penna G, Fibbi B, Amuchastegui S, Cossetti C, Aquilano F, et al. (2009) Human benign prostatic hyperplasia stromal cells as inducers and targets of chronic immuno-mediated inflammation. J Immunol 182: 4056-4064.
5. Purwar R, Werfel T, Wittmann M (2006) IL-13-stimulated human keratinocytes preferentially attract CD4+CCR4+ T cells: possible role in atopic dermatitis. J Invest Dermatol 126: 1043-1051.
6. Sosic D, Richardson JA, Yu K, Ornitz DM, Olson EN (2003) Twist regulates cytokine gene expression through a negative feedback loop that represses NF-kappaB activity. Cell 112: 169-180.
7. Zhou L, Ivanov, II, Spolski R, Min R, Shenderov K, et al. (2007) IL-6 programs T(H)-17 cell differentiation by promoting sequential engagement of the IL-21 and IL-23 pathways. Nat Immunol 8: 967-974.
